# Supplementary material for: Curcumin inhibits type III secretion of Pseudomonas aeruginosa
Source: PeerJ. 2025 Jul 24;13:e19725. doi: 10.7717/peerj.19725 (PMC12296563; doi:10.7717/peerj.19725)
Supplement: Supplemental Information 1 [file peerj-13-19725-s001.pptx]

## Slide 1
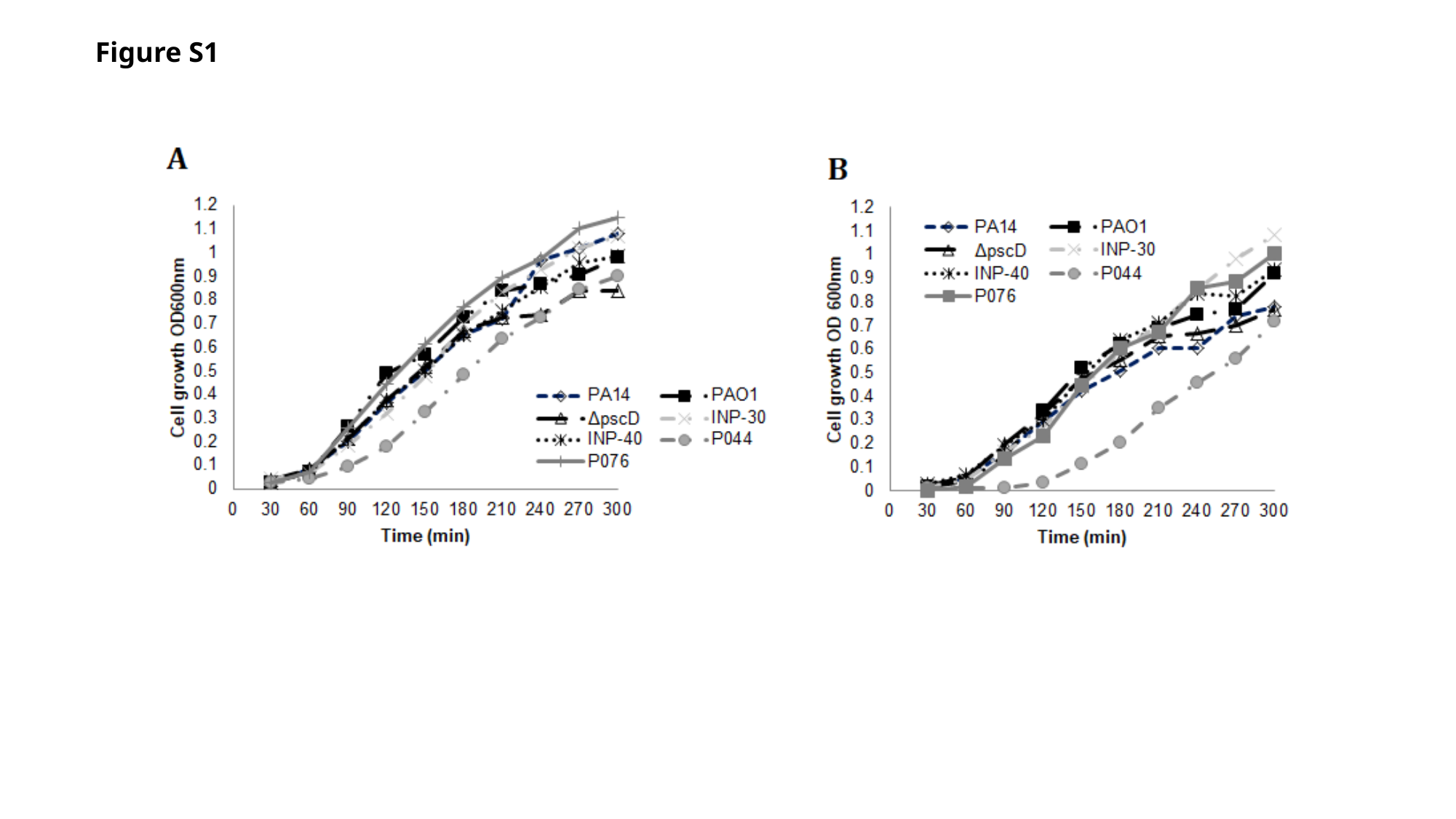

Figure S1

## Slide 2
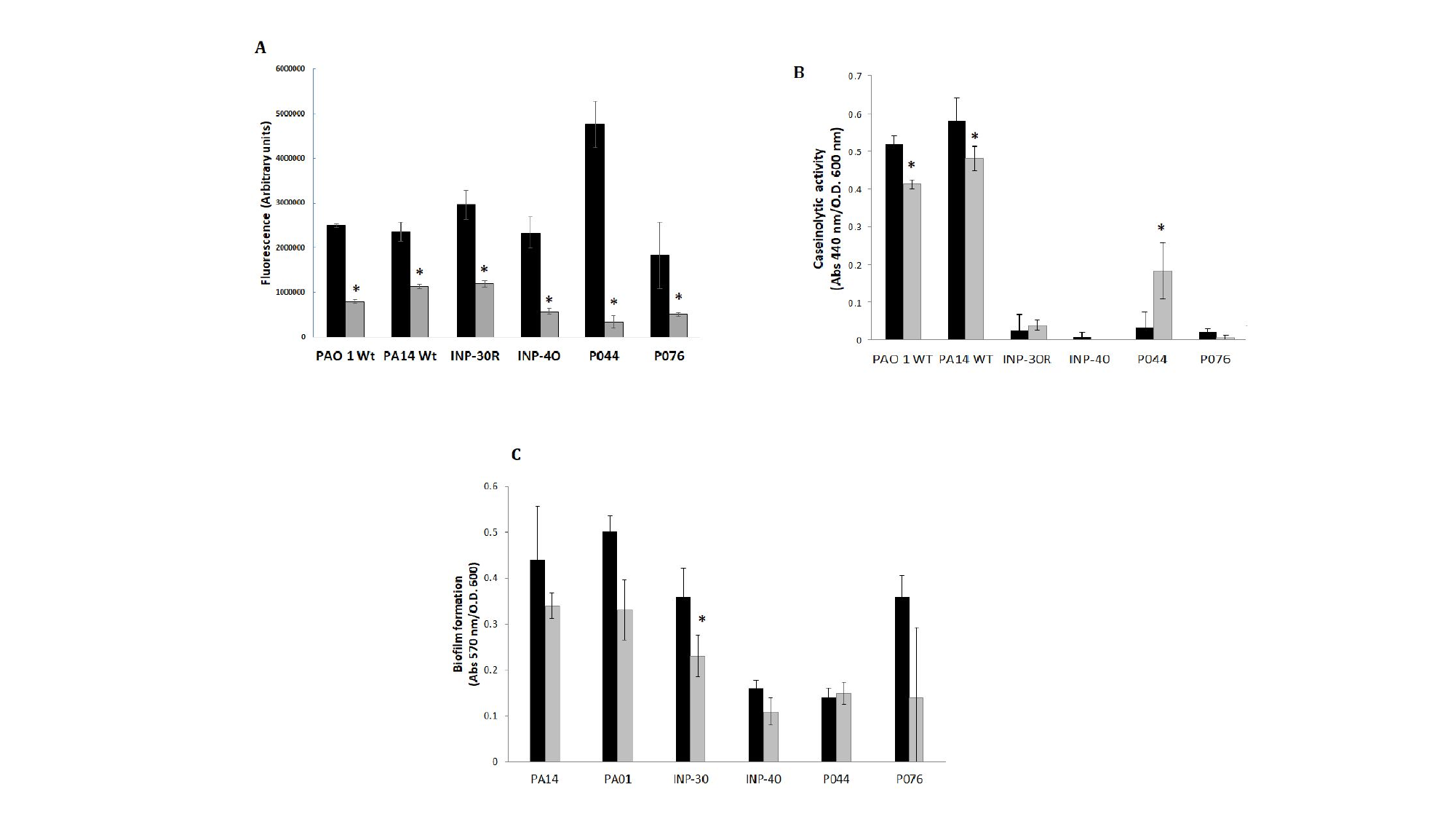

## Slide 3
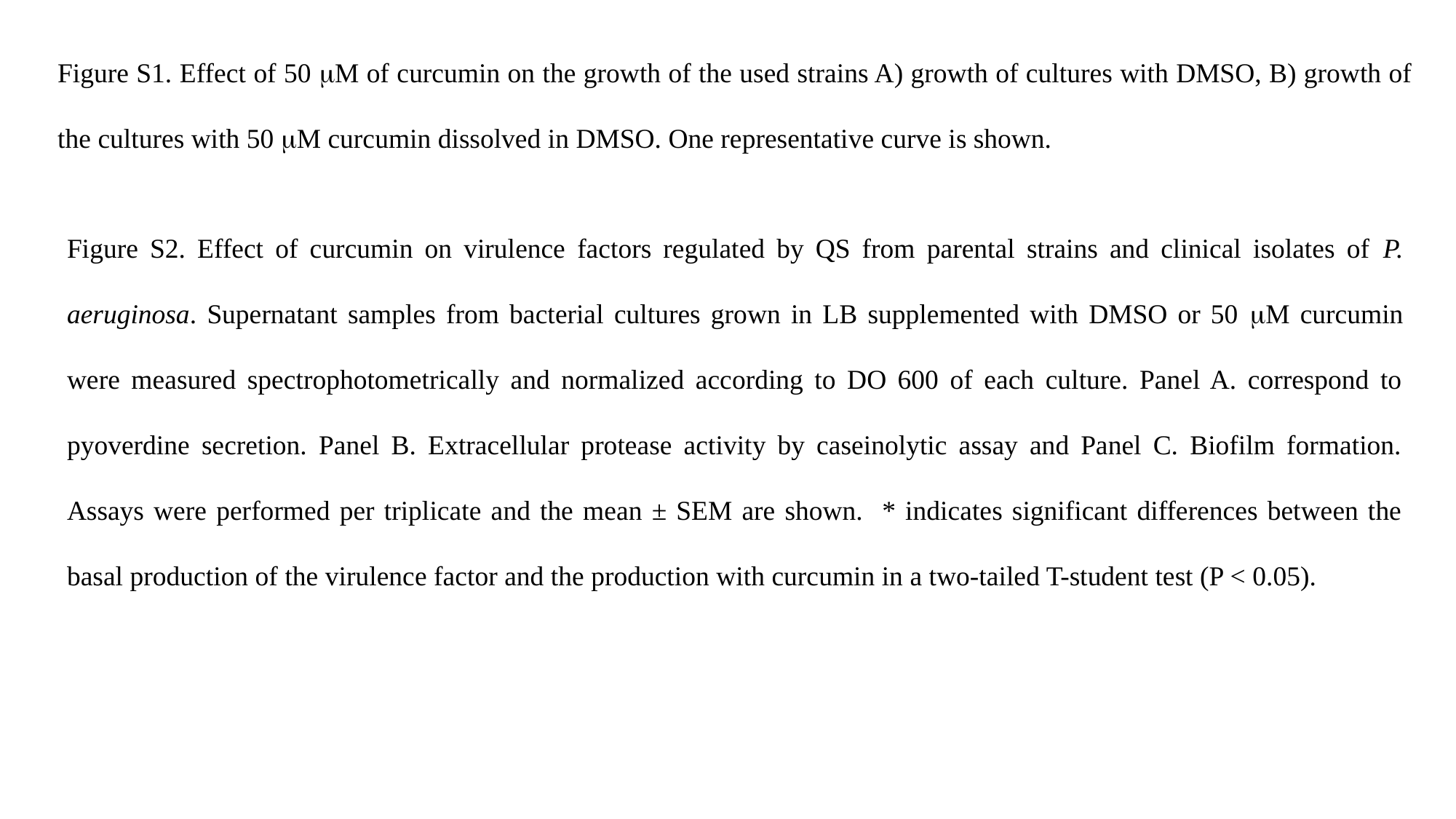

Figure S1. Effect of 50 mM of curcumin on the growth of the used strains A) growth of cultures with DMSO, B) growth of the cultures with 50 mM curcumin dissolved in DMSO. One representative curve is shown.
Figure S2. Effect of curcumin on virulence factors regulated by QS from parental strains and clinical isolates of P. aeruginosa. Supernatant samples from bacterial cultures grown in LB supplemented with DMSO or 50 mM curcumin were measured spectrophotometrically and normalized according to DO 600 of each culture. Panel A. correspond to pyoverdine secretion. Panel B. Extracellular protease activity by caseinolytic assay and Panel C. Biofilm formation. Assays were performed per triplicate and the mean ± SEM are shown. * indicates significant differences between the basal production of the virulence factor and the production with curcumin in a two-tailed T-student test (P < 0.05).
