# Supplementary material for: Curcumin inhibits type III secretion of Pseudomonas aeruginosa
Source: PeerJ. 2025 Jul 24;13:e19725. doi: 10.7717/peerj.19725 (PMC12296563; doi:10.7717/peerj.19725)
Supplement: Supplemental Information 3 [file peerj-13-19725-s003.docx]

Supplementary Table S2. Susceptibility patterns of *Pseudomonas aeruginosa* strains

| Strain | TZP  µg/mL | TZP  Interp. | CAZ  µg/mL | CAZ  Interp. | FEP  µg/mL | FEP  Interp. | ATM  µg/mL | ATM  Interp. | DOR  µg/mL | DOR  Interp. | IMP µg/mL | IMP  Interp. | MEM µg/mL | MEM  Interp. | AK  µg/mL | AK  Interp. | GEN µg/mL | GEN  Interp. | CIP  µg/mL | CIP  Interp. | LVX  µg/mL | LVX  Interp. | CL  µg/mL | CL  Interp. |
| --- | --- | --- | --- | --- | --- | --- | --- | --- | --- | --- | --- | --- | --- | --- | --- | --- | --- | --- | --- | --- | --- | --- | --- | --- |
| INP-40 | 128/4 | R | > 64 | R | 32 | R | 64 | R | 1 | S | 2 | S | 1 | S | 2 | S | 0.5 | S | 1 | I | 1 | S | 0.5 | I |
| P044 | 128/4 | R | > 64 | R | > 64 | R | > 64 | R | 64 | R | 64 | R | 64 | R | 64 | R | > 64 | R | 64 | R | 64 | R | 0.5 | I |
| P076 | 128/4 | R | > 64 | R | > 64 | R | > 64 | R | 8 | R | 8 | R | 4 | I | 64 | R | > 64 | R | 16 | R | 16 | R | 0.5 | I |
| P193 | 8/4 | S | 4 | S | 4 | S | 16 | I | 0.5 | S | 0.5 | S | 2 | S | 1 | S | 0.5 | S | 0.5 | S | 1 | S | 2 | I |
| INP-30R | 64/4 | I | 4 | S | 8 | S | 8 | S | 2 | S | 32 | R | 1 | S | 4 | S | 1 | S | 0.062 | S | 0.062 | S | 0.25 | I |

TZP, Piperacillin/Tazobactam; CAZ, Ceftazidime; FEP, Cefepime; ATM, Aztreonam; DOR, Doripenem; IMP, Imipenem; MEM, Meropenem AK, Amikacin; GEN, Gentamicin; CIP, Ciprofloxacin LVX, Levofloxacin CL, Colistin; INTERP, Interpretation; S, Susceptible; R, Resistant and I, Intermediate.

Breakpoints CLSI M100 Ed32, 2022 (Clinical and Laboratory Standards Institute 2022) y (CLSI 2018)

Clinical and Laboratory Standards Institute. 2022. “M100. Performance Standards for Antimicrobial Susceptibility Testing; Approved Guideline. 32th Ed.” 2022. 2022.

CLSI. 2018. “M07 Methods for Dilution Antimicrobial Susceptibility Tests for Bacteria That Grow Aerobically, 11th Edition.” CLSI.
