# Supplementary material for: Curcumin inhibits type III secretion of Pseudomonas aeruginosa
Source: PeerJ. 2025 Jul 24;13:e19725. doi: 10.7717/peerj.19725 (PMC12296563; doi:10.7717/peerj.19725)
Supplement: Supplemental Information 4 [file peerj-13-19725-s004.zip › crude data and blots/Figure 1/Figure 1.pptx]

## Slide 1
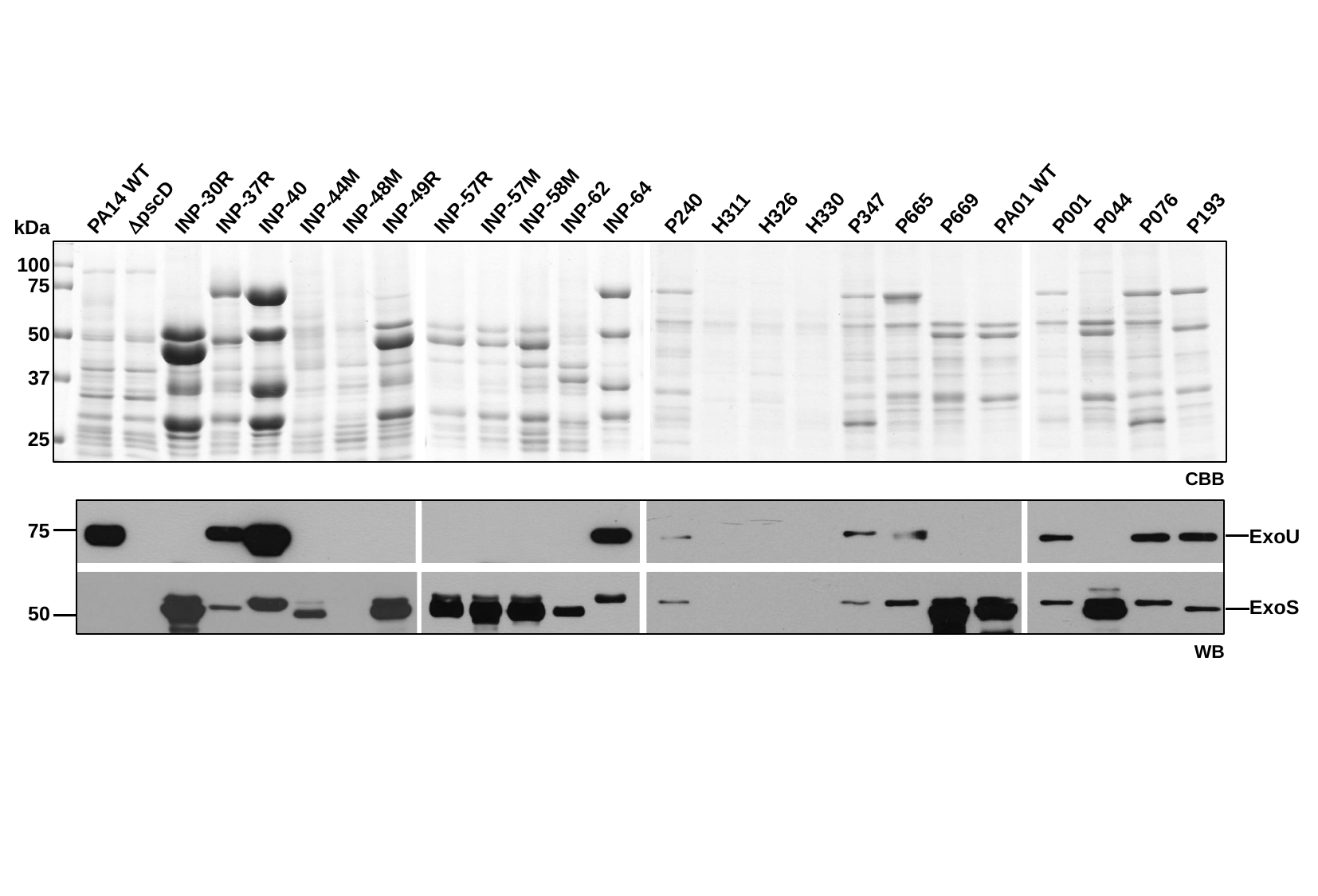

INP-57M
INP-49R
INP-40
P001
P044
P076
INP-44M
INP-64
PA01 WT
P347
P665
P669
INP-48M
P193
INP-58M
INP-30R
INP-57R
H326
INP-37R
INP-62
H330
PA14 WT
DpscD
H311
P240
kDa
100
75
50
37
25
CBB
75
ExoU
ExoS
50
WB

## Slide 2
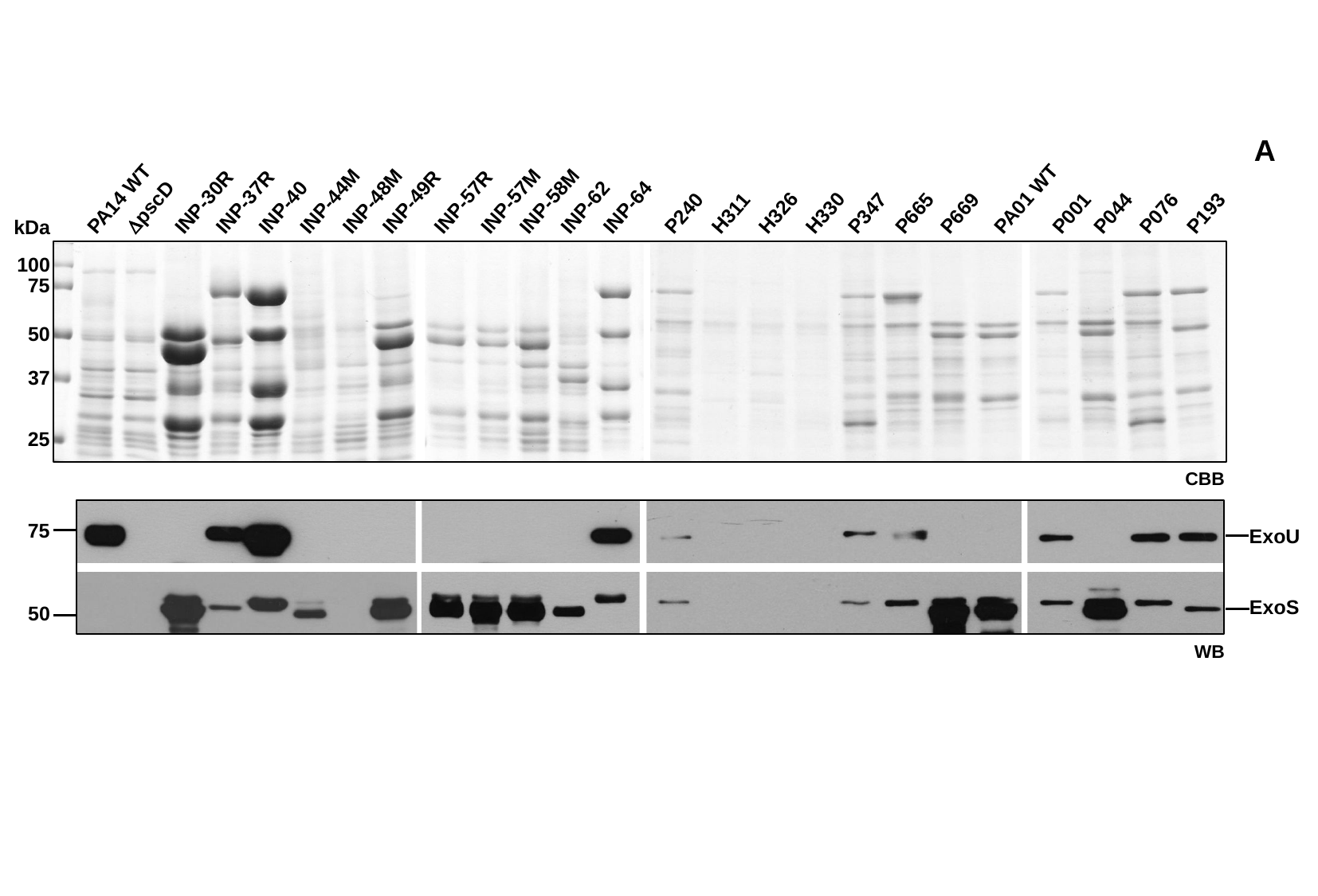

A
INP-57M
INP-49R
INP-40
P001
P044
P076
INP-44M
INP-64
PA01 WT
P347
P665
P669
INP-48M
P193
INP-58M
INP-30R
INP-57R
H326
INP-37R
INP-62
H330
PA14 WT
DpscD
H311
P240
kDa
100
75
50
37
25
CBB
75
ExoU
ExoS
50
WB
