# Supplementary material for: Curcumin inhibits type III secretion of Pseudomonas aeruginosa
Source: PeerJ. 2025 Jul 24;13:e19725. doi: 10.7717/peerj.19725 (PMC12296563; doi:10.7717/peerj.19725)
Supplement: Supplemental Information 4 [file peerj-13-19725-s004.zip › crude data and blots/Figure 2/Figure 2.pptx]

## Slide 1
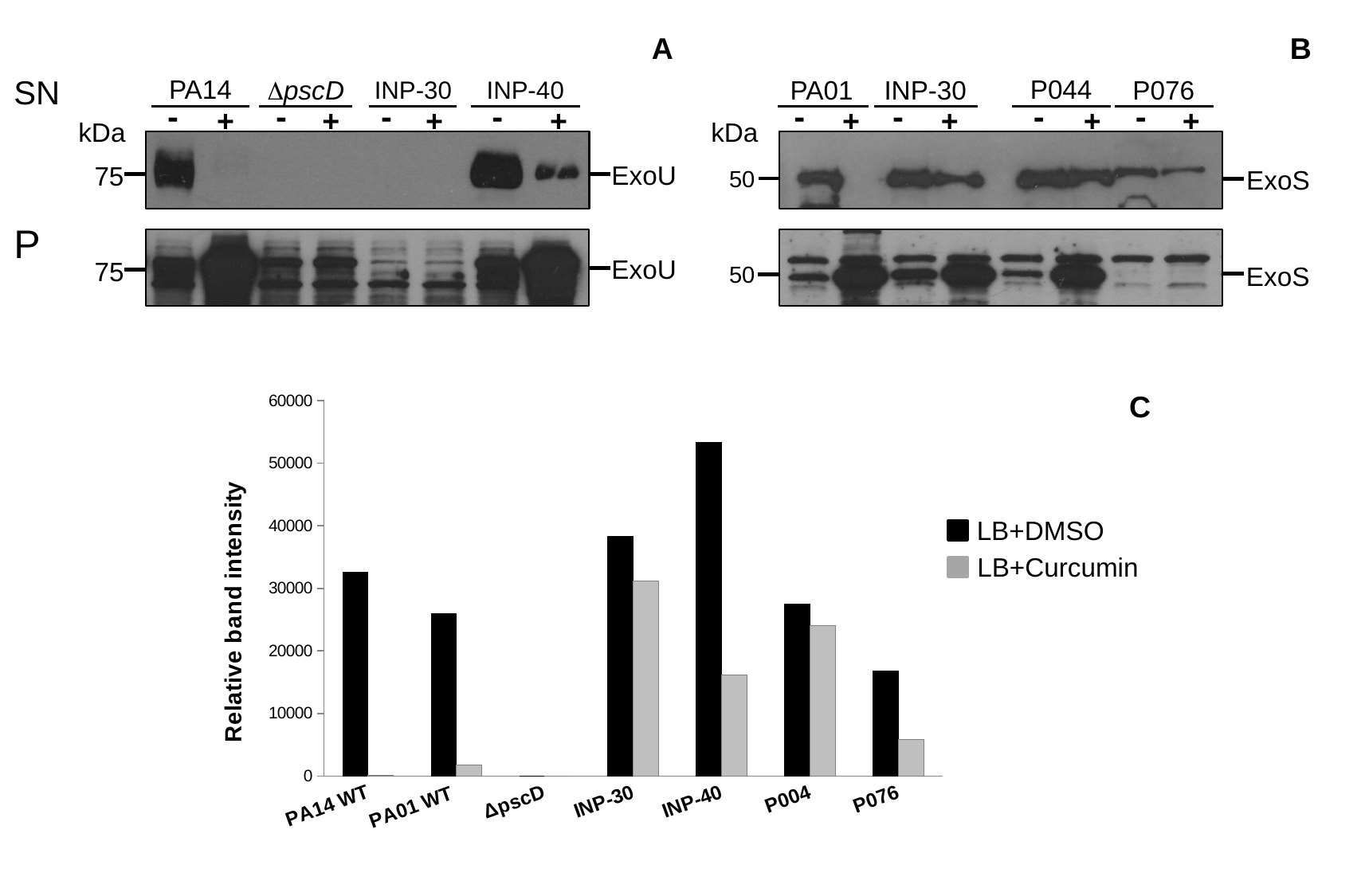

A
B
SN
P044
PA14
DpscD
PA01
INP-30
P076
INP-30
INP-40
-
-
-
-
-
-
-
-
+
+
+
+
+
+
+
+
kDa
kDa
ExoU
75
ExoS
50
P
ExoU
75
50
ExoS
### Chart
| Category | LB | Curcumina |
|---|---|---|
| PA14 WT | 32561.06466666667 | 86.89266666666667 |
| PA01 WT | 25982.108999999997 | 1781.6316666666664 |
| ΔpscD | 0.0 | 0.0 |
| INP-30 | 38267.95133333333 | 31205.519 |
| INP-40 | 53287.943999999996 | 16113.543 |
| P004 | 27440.54533333333 | 24078.410333333333 |
| P076 | 16801.630999999998 | 5803.559 |C
LB+DMSO
LB+Curcumin

## Slide 2
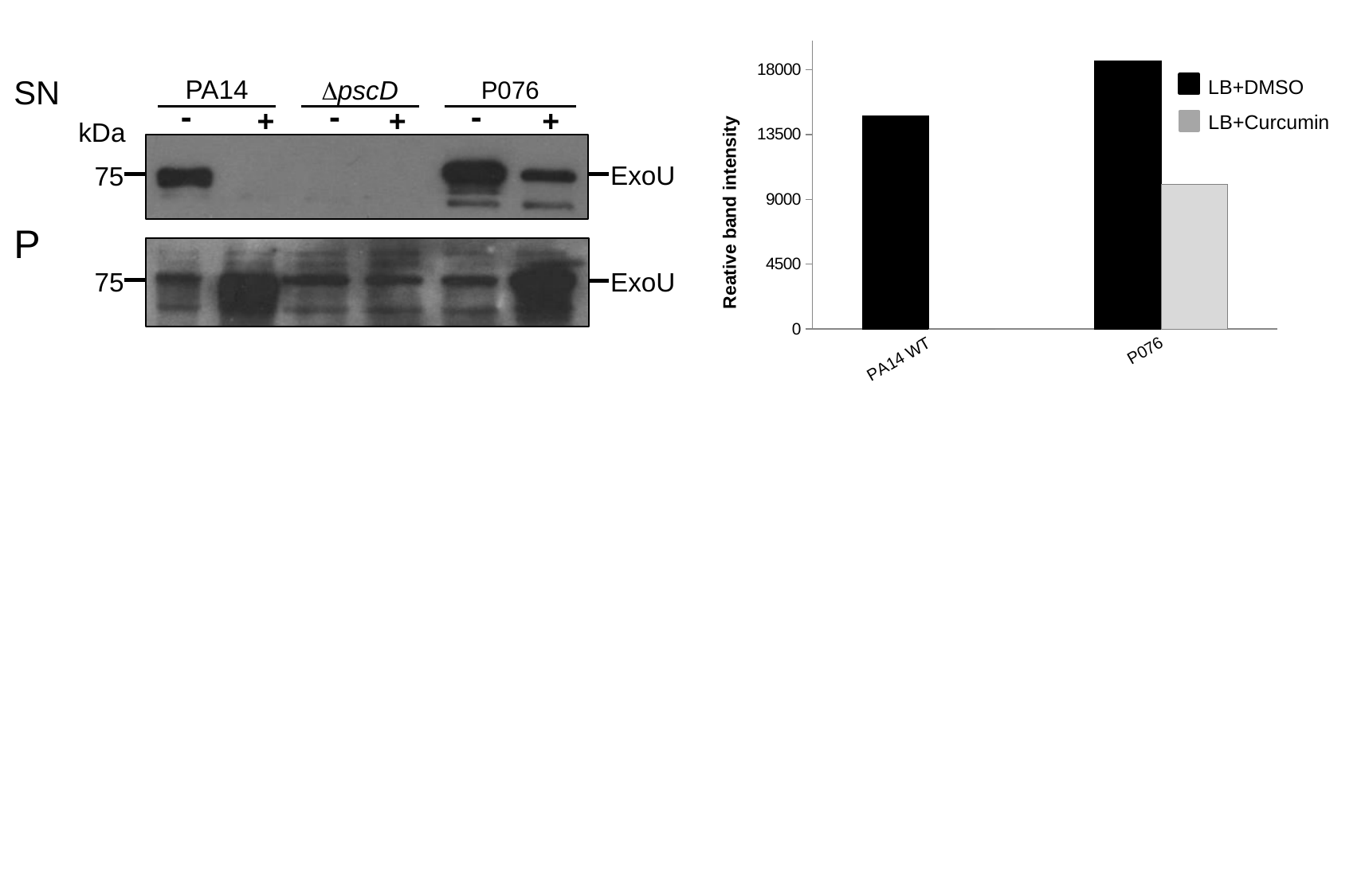

### Chart
| Category | LB | Curcumin |
|---|---|---|
| PA14 WT | 14779.295666666667 | 0.0 |
| P076 | 18606.981 | 10021.300333333333 |LB+DMSO
SN
PA14
DpscD
P076
-
-
-
+
+
+
LB+Curcumin
kDa
ExoU
75
P
75
ExoU

## Slide 3
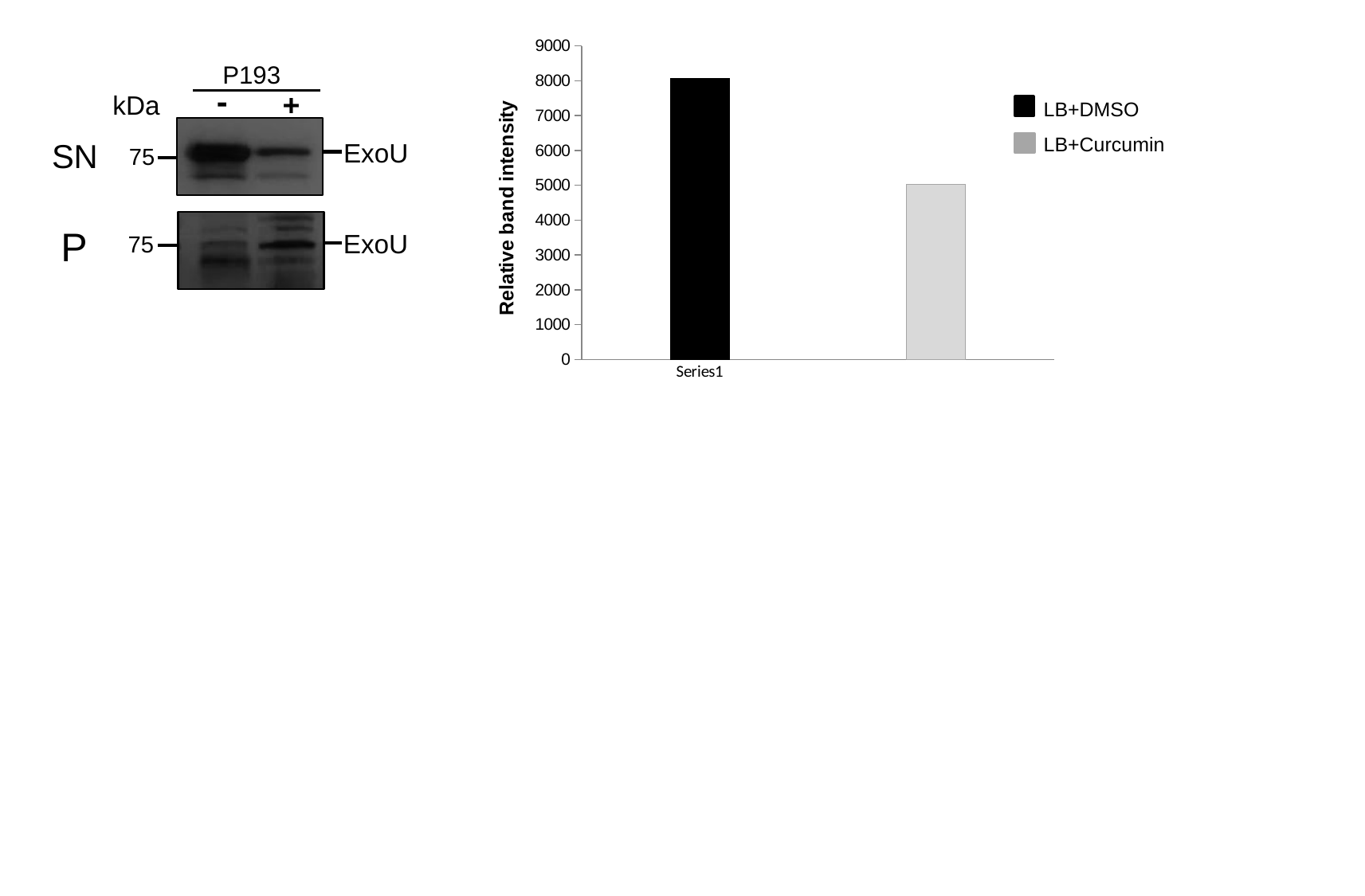

### Chart
| Category | P193 |
|---|---|
| | 8063.739333333334 |
| | 5020.904666666666 |
P193
-
+
kDa
LB+DMSO
LB+Curcumin
SN
ExoU
75
P
ExoU
75
