# Supplementary material for: Curcumin inhibits type III secretion of Pseudomonas aeruginosa
Source: PeerJ. 2025 Jul 24;13:e19725. doi: 10.7717/peerj.19725 (PMC12296563; doi:10.7717/peerj.19725)
Supplement: Supplemental Information 4 [file peerj-13-19725-s004.zip › crude data and blots/Figure S2/Discard/Figure 3.pptx]

## Slide 1
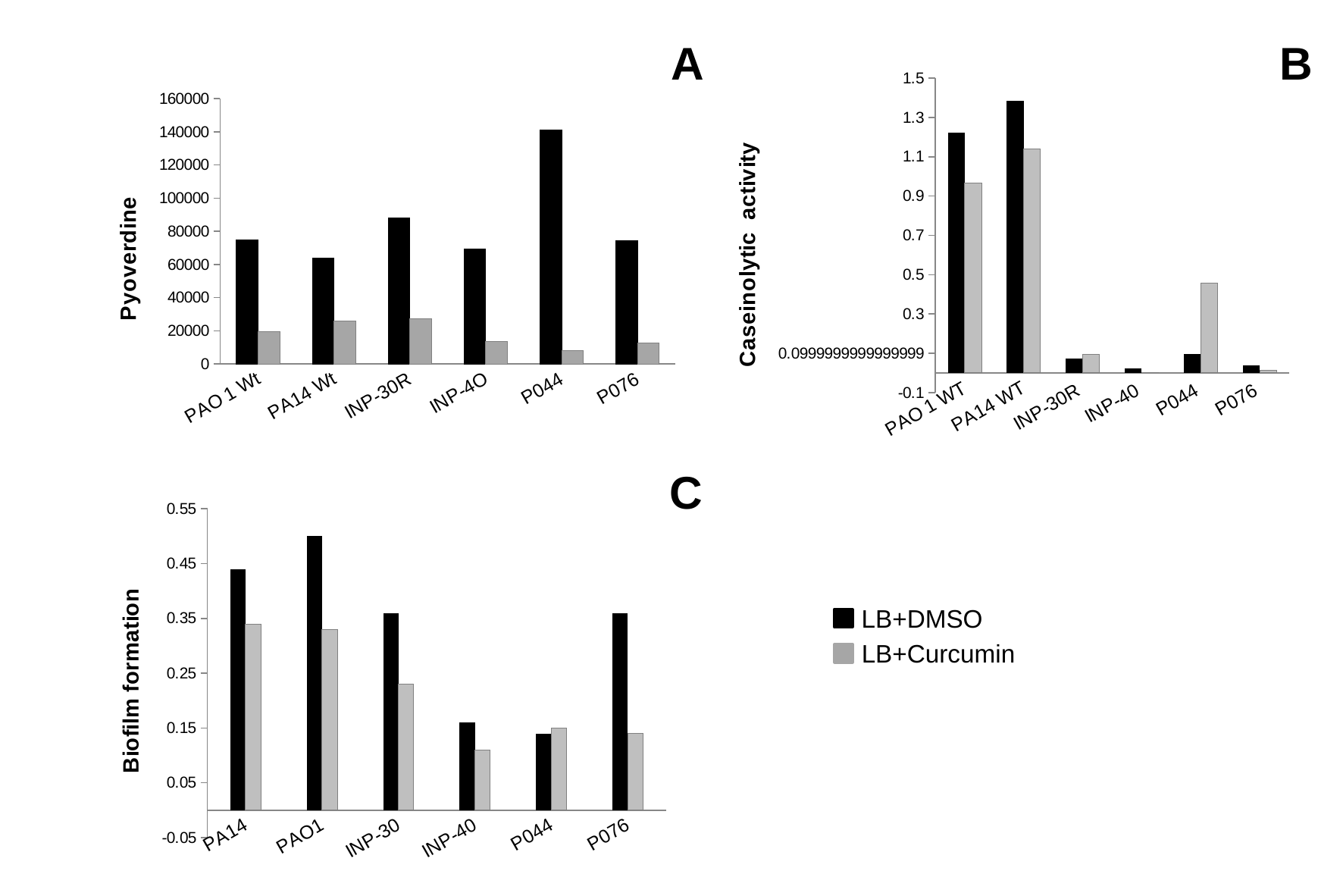

A
B
### Chart
| Category | No curcumina | 50 μM curcuma |
|---|---|---|
| PAO 1 WT | 1.2212255444813584 | 0.9676434676434676 |
| PA14 WT | 1.3845076060848678 | 1.140746819860484 |
| INP-30R | 0.07434262948207172 | 0.09626078619367208 |
| INP-40 | 0.0213957375679064 | 0.0 |
| P044 | 0.09503637141634573 | 0.45635052689961164 |
| P076 | 0.038709677419354854 | 0.015133531157270032 |
### Chart
| Category | LB | Curcumin 50 μM |
|---|---|---|
| PAO 1 Wt | 74782.39 | 19443.13 |
| PA14 Wt | 63746.29 | 25991.41 |
| INP-30R | 88137.3 | 27007.44 |
| INP-4O | 69285.83 | 13426.42 |
| P044 | 141276.28 | 8047.58 |
| P076 | 74143.56 | 12630.26 |C
### Chart
| Category | LB | 50 μM Curcuma |
|---|---|---|
| PA14 | 0.44 | 0.34 |
| PAO1 | 0.5 | 0.33 |
| INP-30 | 0.36 | 0.23 |
| INP-40 | 0.16 | 0.11 |
| P044 | 0.14 | 0.15 |
| P076 | 0.36 | 0.14 |LB+DMSO
LB+Curcumin
