# Supplementary material for: Curcumin inhibits type III secretion of Pseudomonas aeruginosa
Source: PeerJ. 2025 Jul 24;13:e19725. doi: 10.7717/peerj.19725 (PMC12296563; doi:10.7717/peerj.19725)
Supplement: Supplemental Information 4 [file peerj-13-19725-s004.zip › crude data and blots/Figure S2/Figure 3.Final.pptx]

## Slide 1
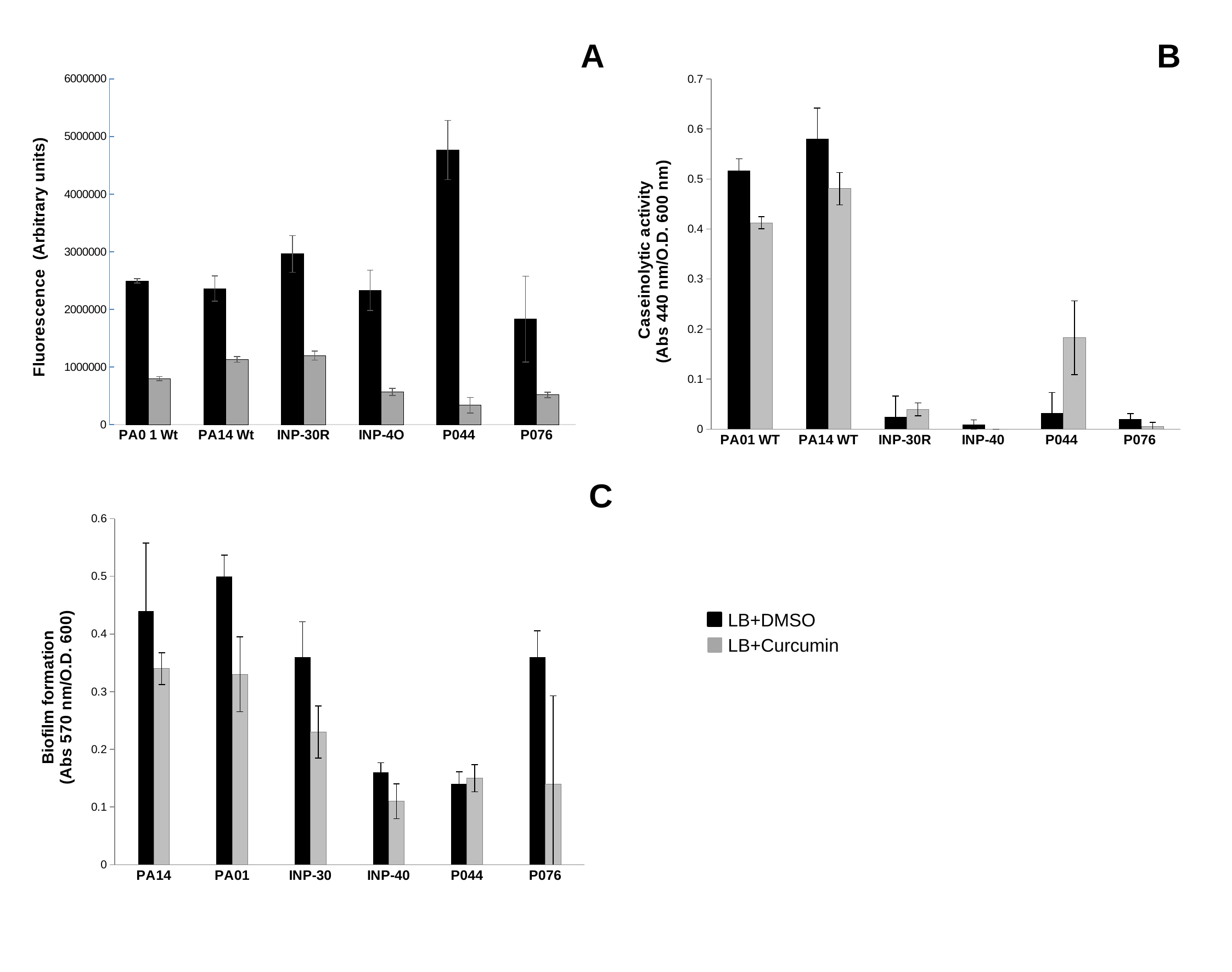

A
B
### Chart
| Category | LB | Curcumin 50 μM |
|---|---|---|
| PA0 1 Wt | 2492746.400885936 | 796615.0989153172 |
| PA14 Wt | 2360674.69879518 | 1129112.757201646 |
| INP-30R | 2961507.6305220886 | 1195180.1932367152 |
| INP-4O | 2331940.928270042 | 566299.5614035088 |
| P044 | 4764251.948051948 | 334758.7354409318 |
| P076 | 1830300.0 | 514955.984174085 |
### Chart
| Category | No curcumina | 50 μM curcuma |
|---|---|---|
| PA01 WT | 0.5165374677002583 | 0.41257631257631266 |
| PA14 WT | 0.5802642113690953 | 0.48067295855560105 |
| INP-30R | 0.02478087649402391 | 0.039597315436241606 |
| INP-40 | 0.009611366485582954 | 0.0 |
| P044 | 0.032563115104835255 | 0.1825845812534664 |
| P076 | 0.02000000000000001 | 0.005044510385756678 |C
### Chart
| Category | LB | 50 μM Curcuma |
|---|---|---|
| PA14 | 0.44 | 0.34 |
| PA01 | 0.5 | 0.33 |
| INP-30 | 0.36 | 0.23 |
| INP-40 | 0.16 | 0.11 |
| P044 | 0.14 | 0.15 |
| P076 | 0.36 | 0.14 |LB+DMSO
LB+Curcumin
