# Supplementary figures and images for: Curcumin inhibits type III secretion of Pseudomonas aeruginosa
Source: PeerJ. 2025 Jul 24;13:e19725. doi: 10.7717/peerj.19725 (PMC12296563; doi:10.7717/peerj.19725)

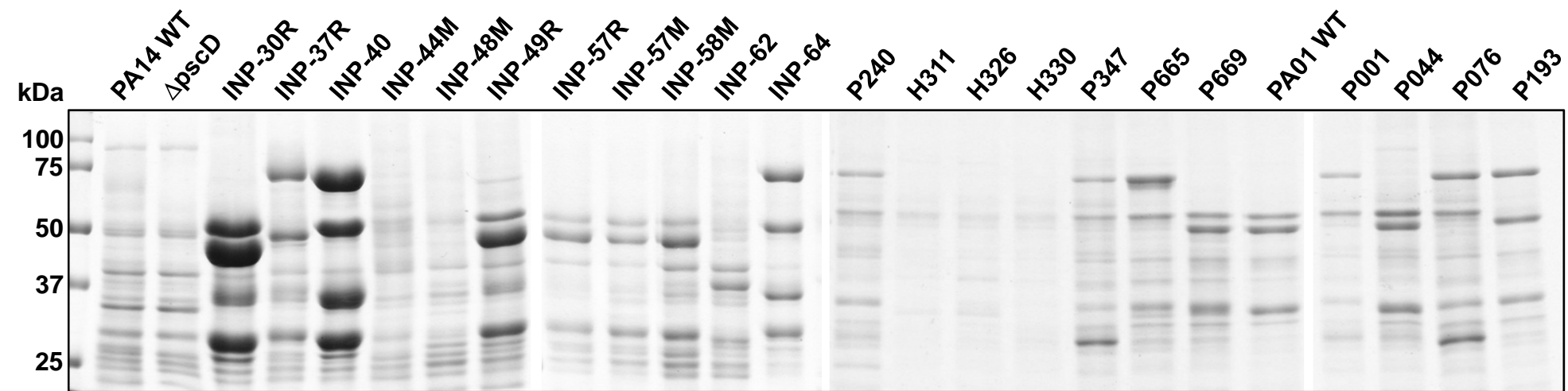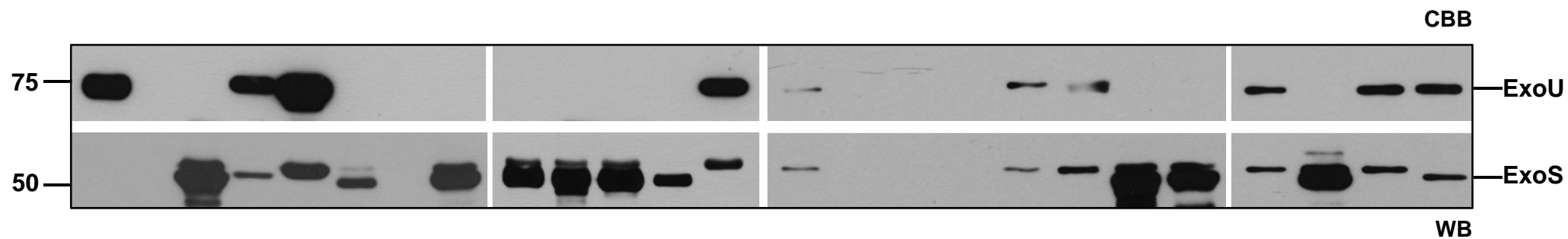

Supplement: Supplemental Information 4 [file peerj-13-19725-s004.zip › crude data and blots/Figure 1/Figure 1.pdf]

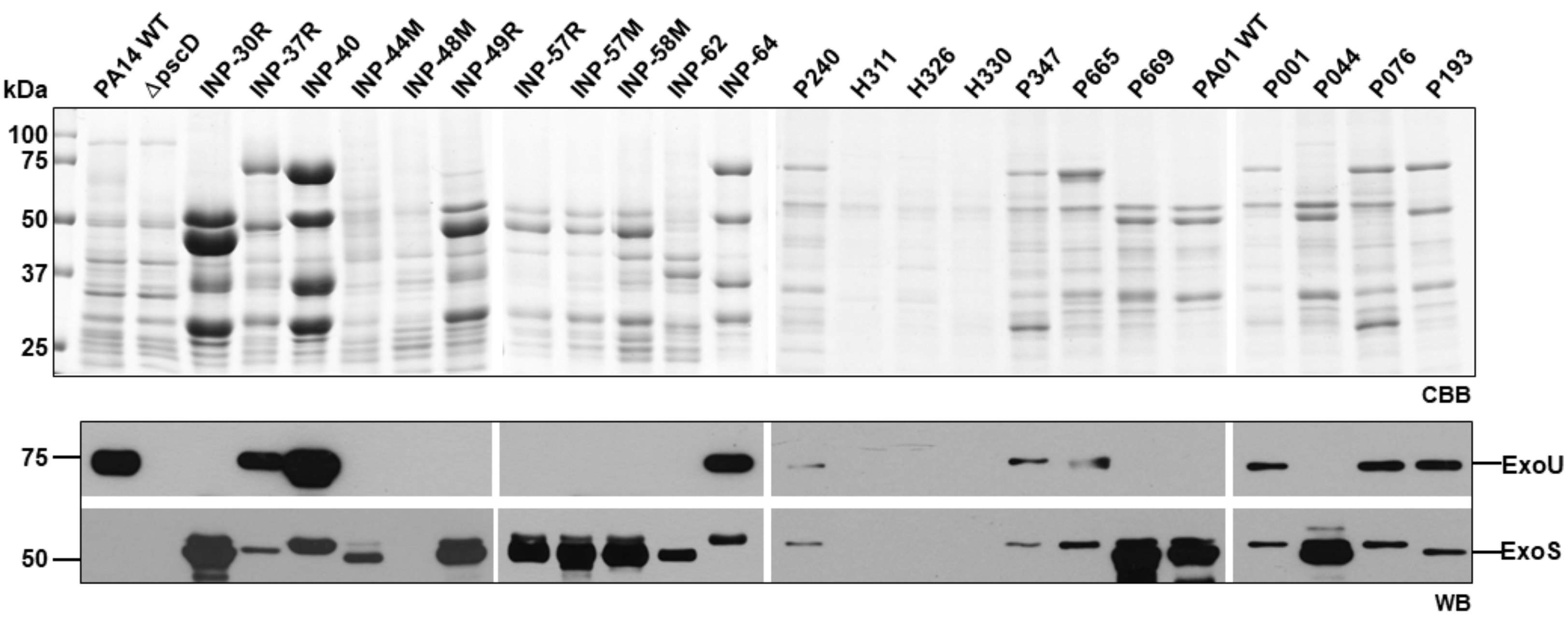

Supplement: Supplemental Information 4 [file peerj-13-19725-s004.zip › crude data and blots/Figure 1/Figure 1.tiff]

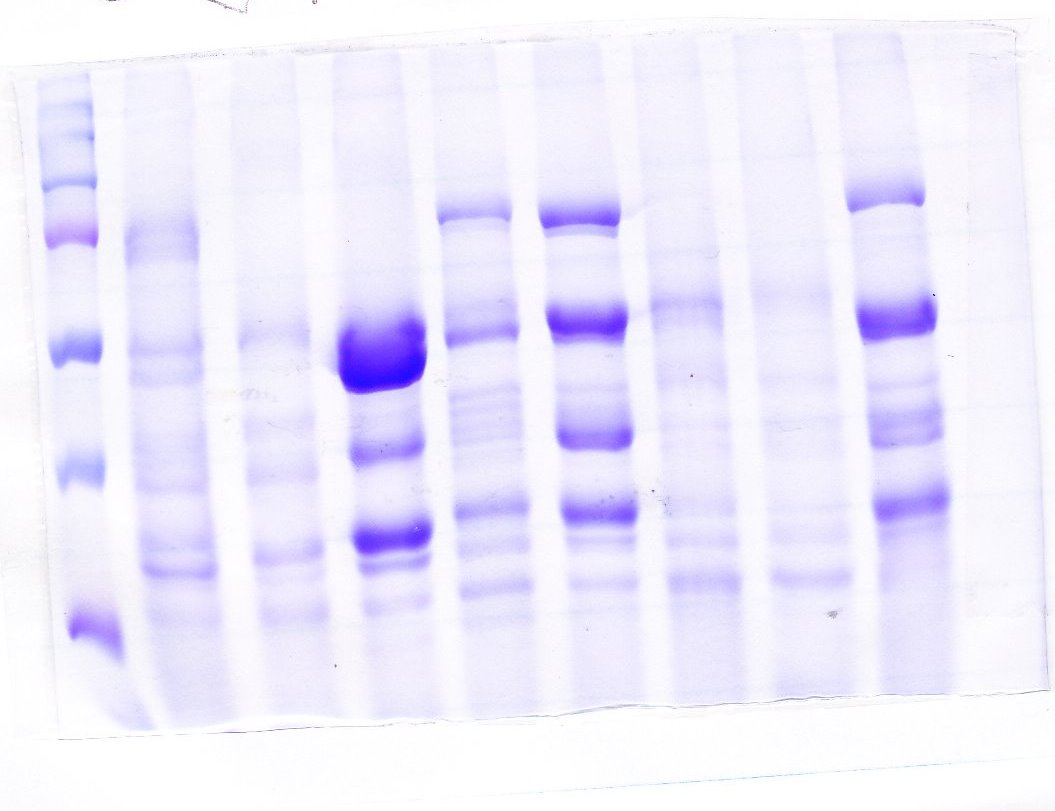

Supplement: Supplemental Information 4 [file peerj-13-19725-s004.zip › crude data and blots/Figure 1/Original blots/Duplicados/CBB Fig 1.jpg]

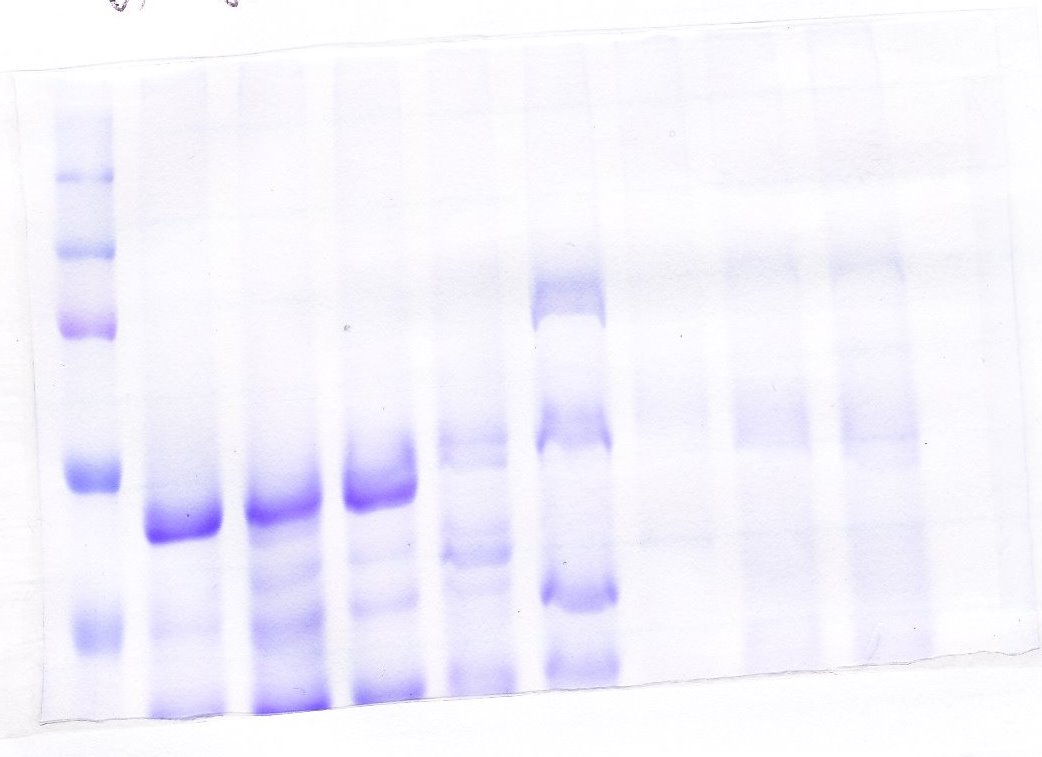

Supplement: Supplemental Information 4 [file peerj-13-19725-s004.zip › crude data and blots/Figure 1/Original blots/Duplicados/CBB Fig 2.jpg]

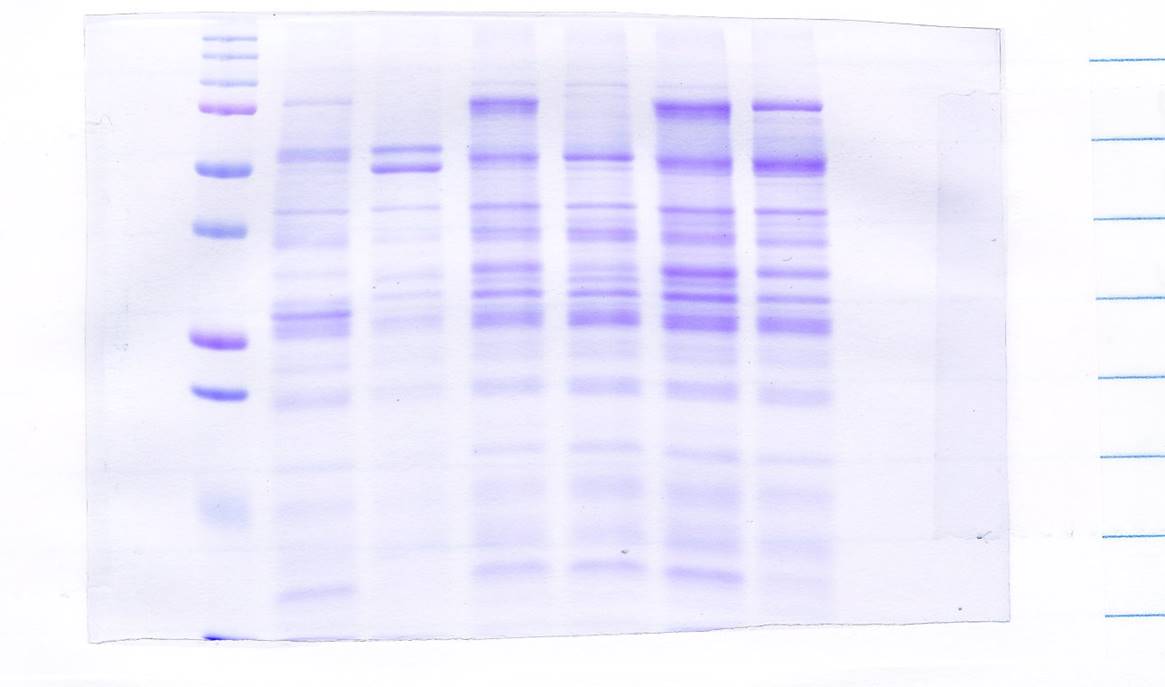

Supplement: Supplemental Information 4 [file peerj-13-19725-s004.zip › crude data and blots/Figure 1/Original blots/Duplicados/CBB Fig 4.jpg]

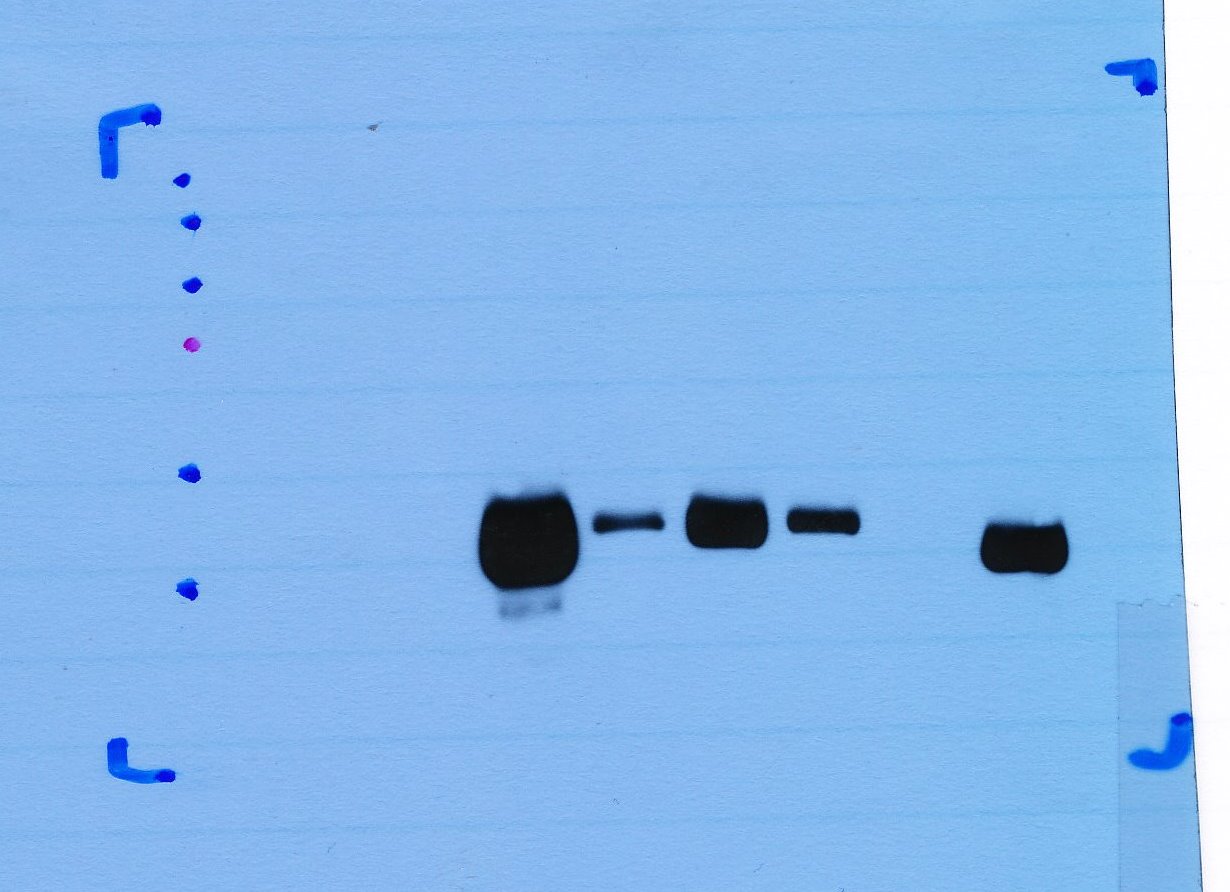

Supplement: Supplemental Information 4 [file peerj-13-19725-s004.zip › crude data and blots/Figure 1/Original blots/Duplicados/WB EXoS Fig 1.1.jpg]

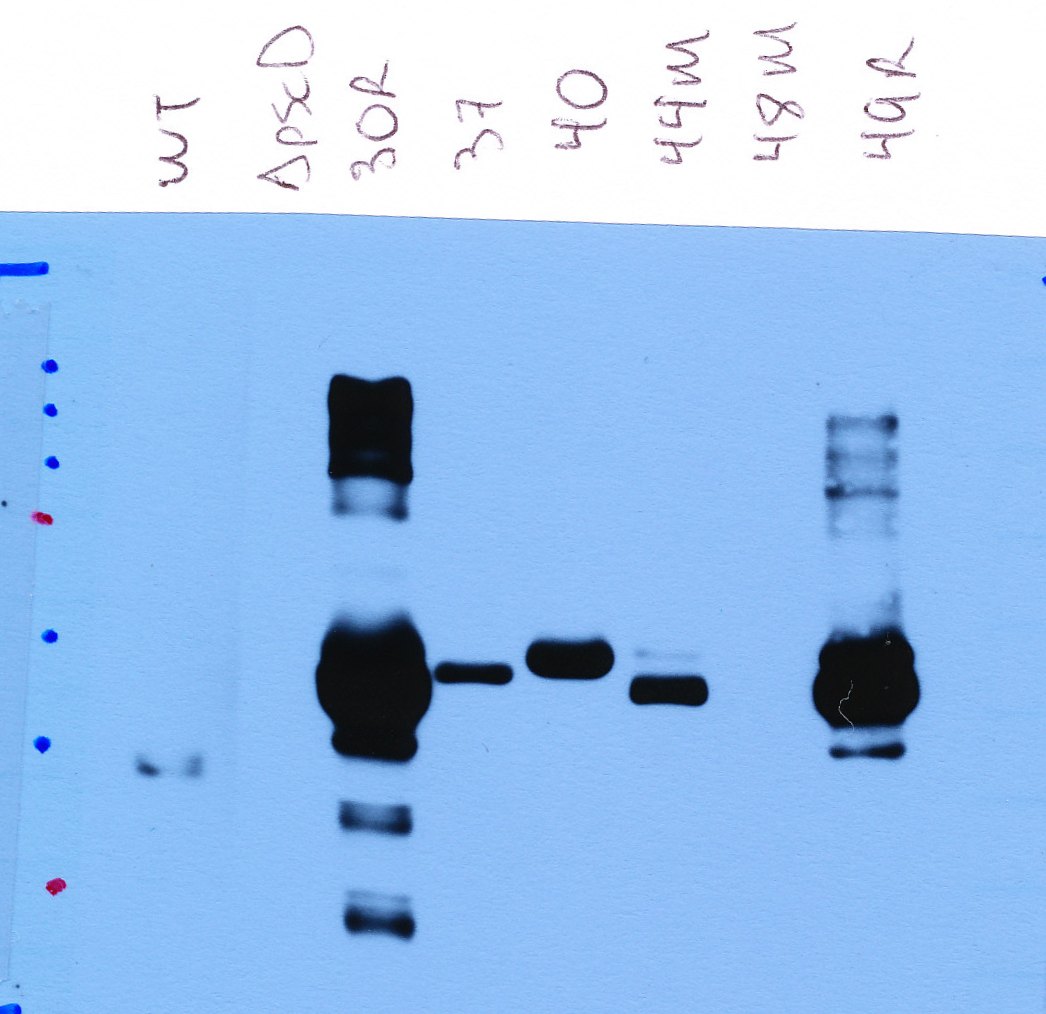

Supplement: Supplemental Information 4 [file peerj-13-19725-s004.zip › crude data and blots/Figure 1/Original blots/Duplicados/WB ExoS Fig 1.2.jpg]

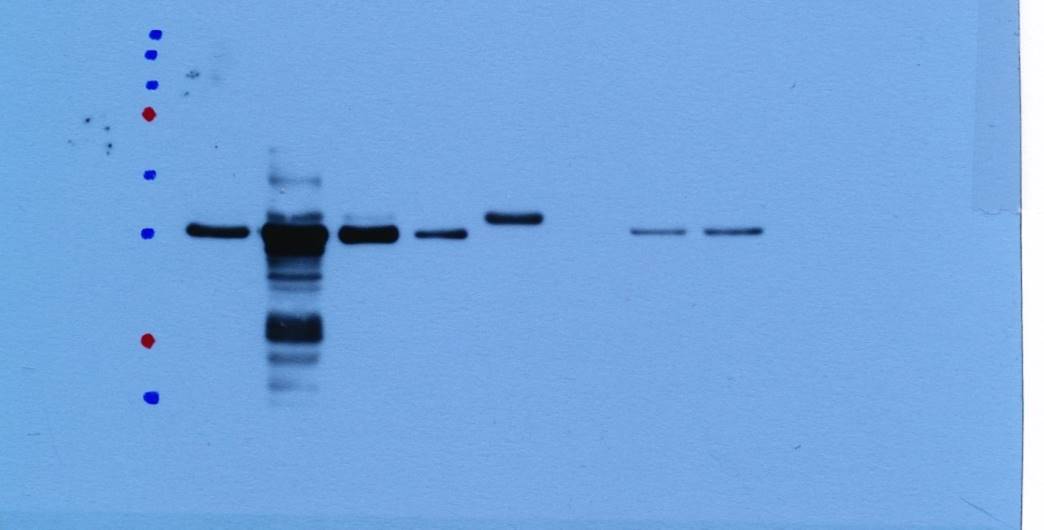

Supplement: Supplemental Information 4 [file peerj-13-19725-s004.zip › crude data and blots/Figure 1/Original blots/Duplicados/WB EXoS Fig 2.1.jpg]

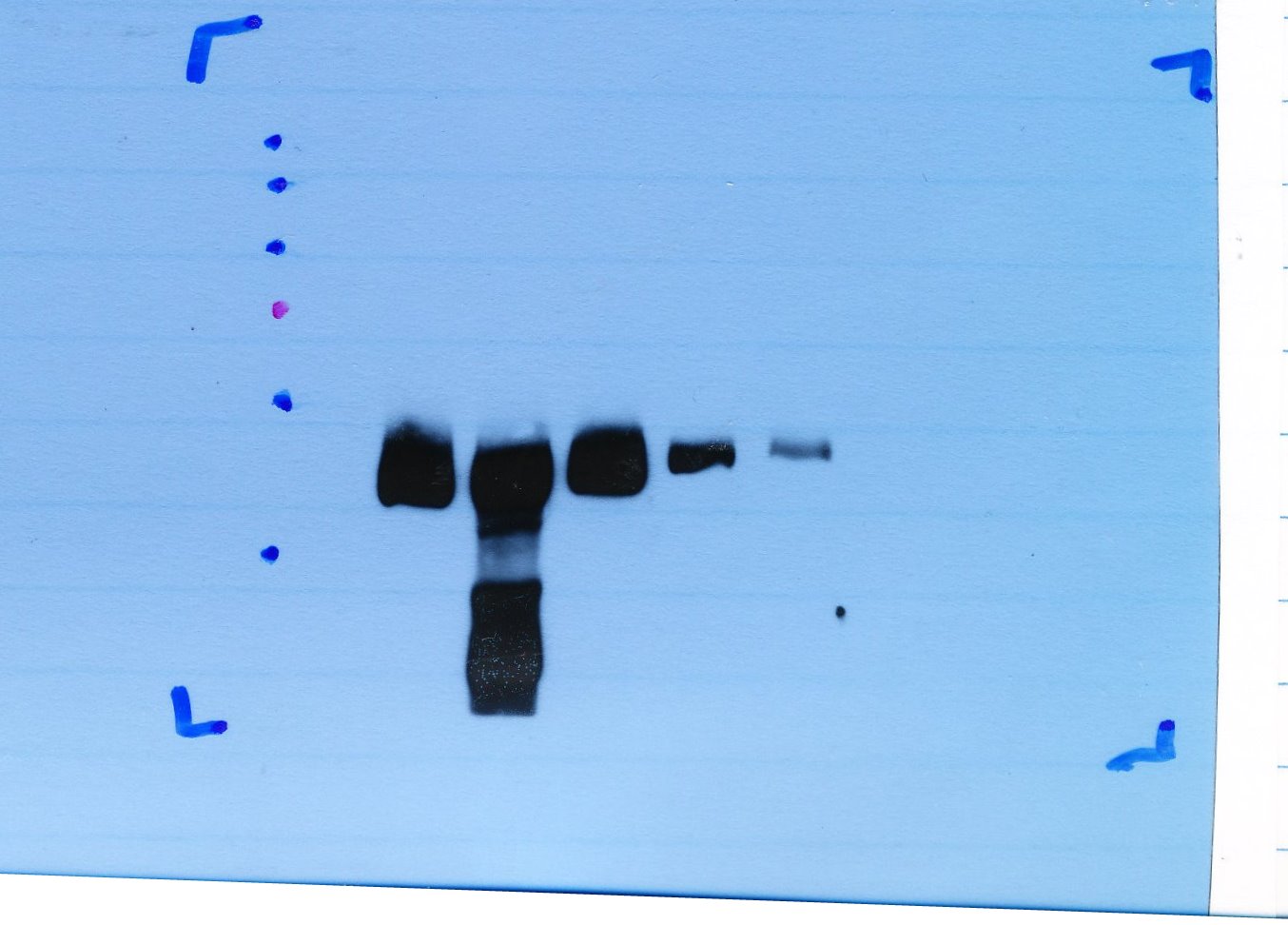

Supplement: Supplemental Information 4 [file peerj-13-19725-s004.zip › crude data and blots/Figure 1/Original blots/Duplicados/WB EXoS Fig 2.2.jpg]

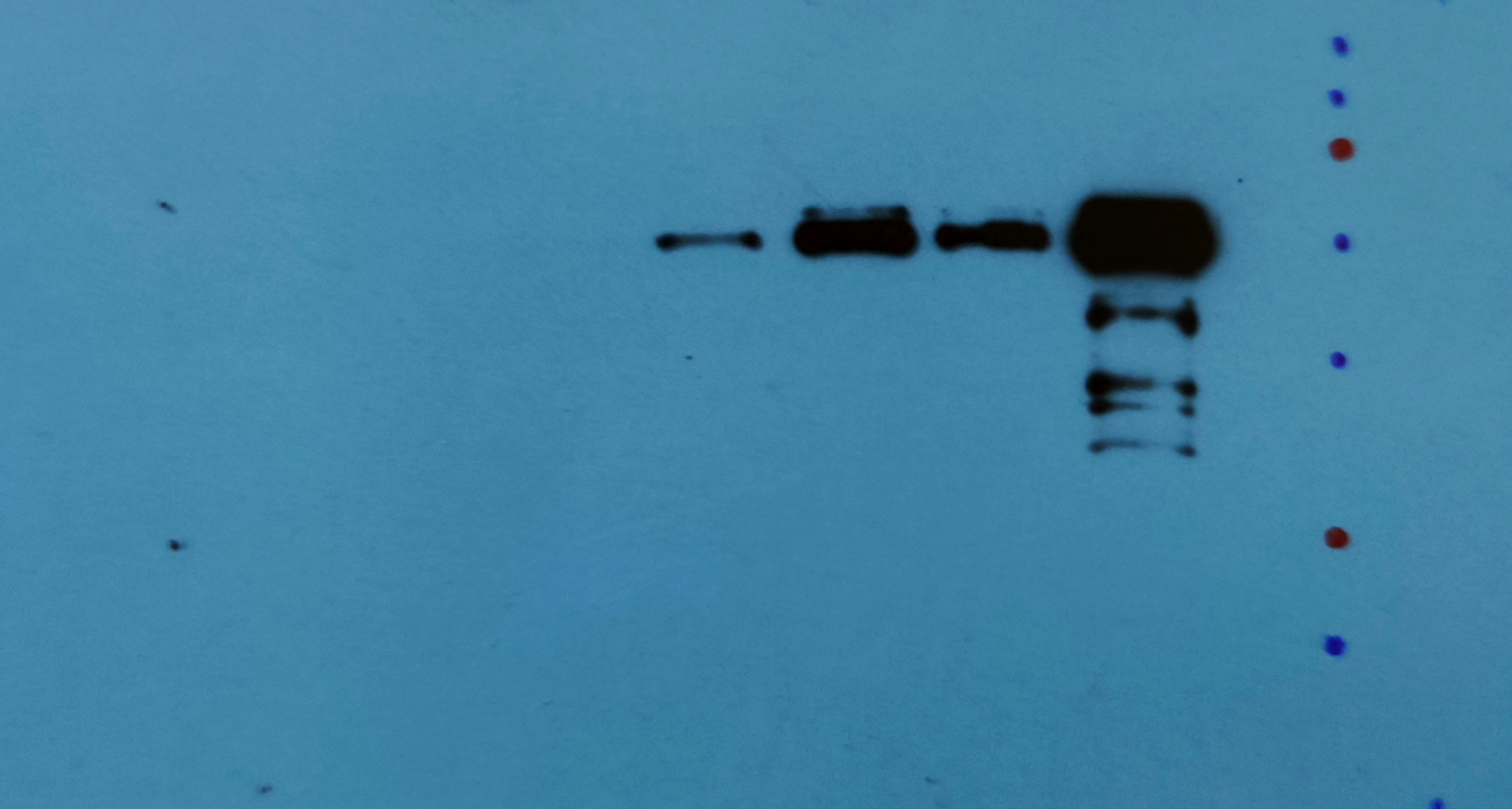

Supplement: Supplemental Information 4 [file peerj-13-19725-s004.zip › crude data and blots/Figure 1/Original blots/Duplicados/WB ExoS Fig 3.1.jpg]

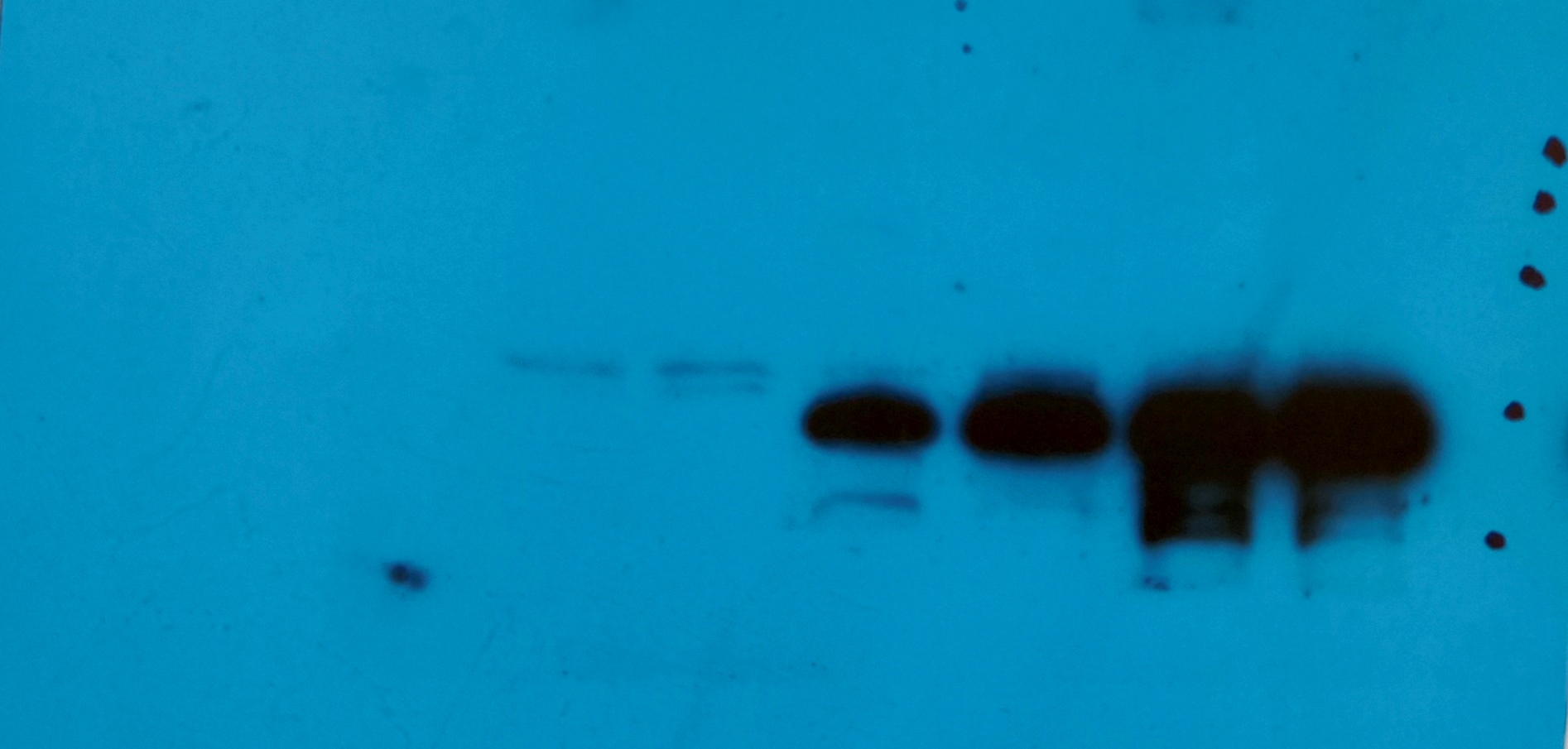

Supplement: Supplemental Information 4 [file peerj-13-19725-s004.zip › crude data and blots/Figure 1/Original blots/Duplicados/WB ExoS Fig 3.2.jpg]

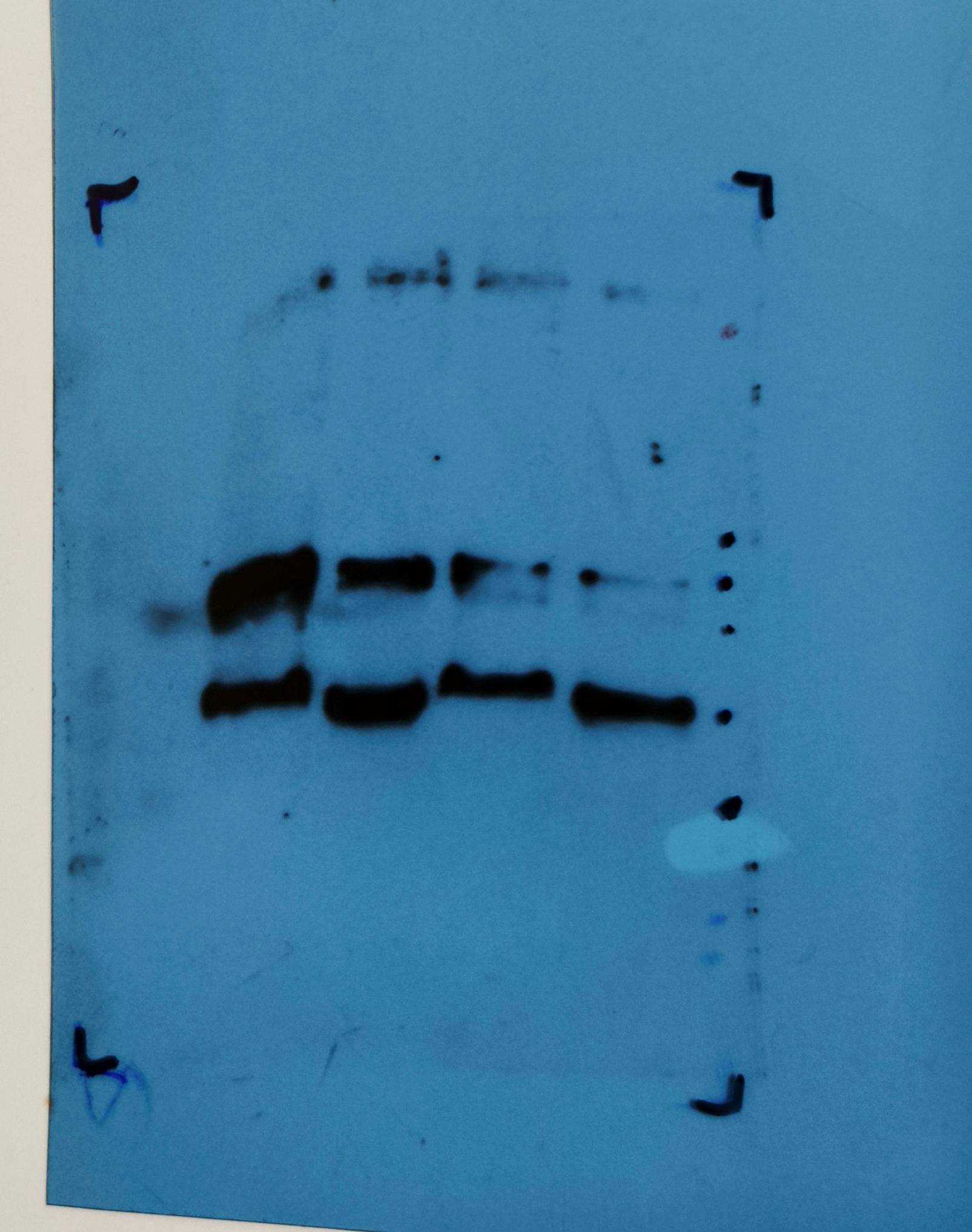

Supplement: Supplemental Information 4 [file peerj-13-19725-s004.zip › crude data and blots/Figure 1/Original blots/Duplicados/WB ExoS Fig 4.1.jpg]

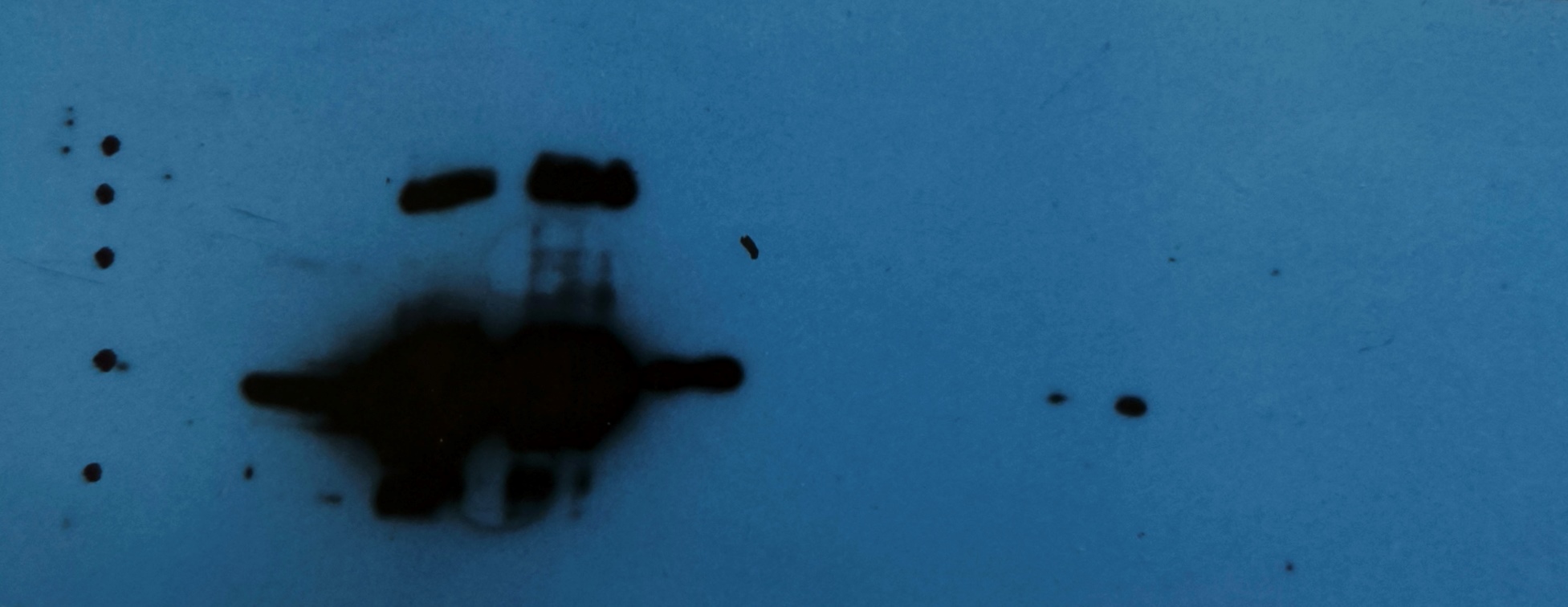

Supplement: Supplemental Information 4 [file peerj-13-19725-s004.zip › crude data and blots/Figure 1/Original blots/Duplicados/WB ExoS Fig 4.2.jpg]

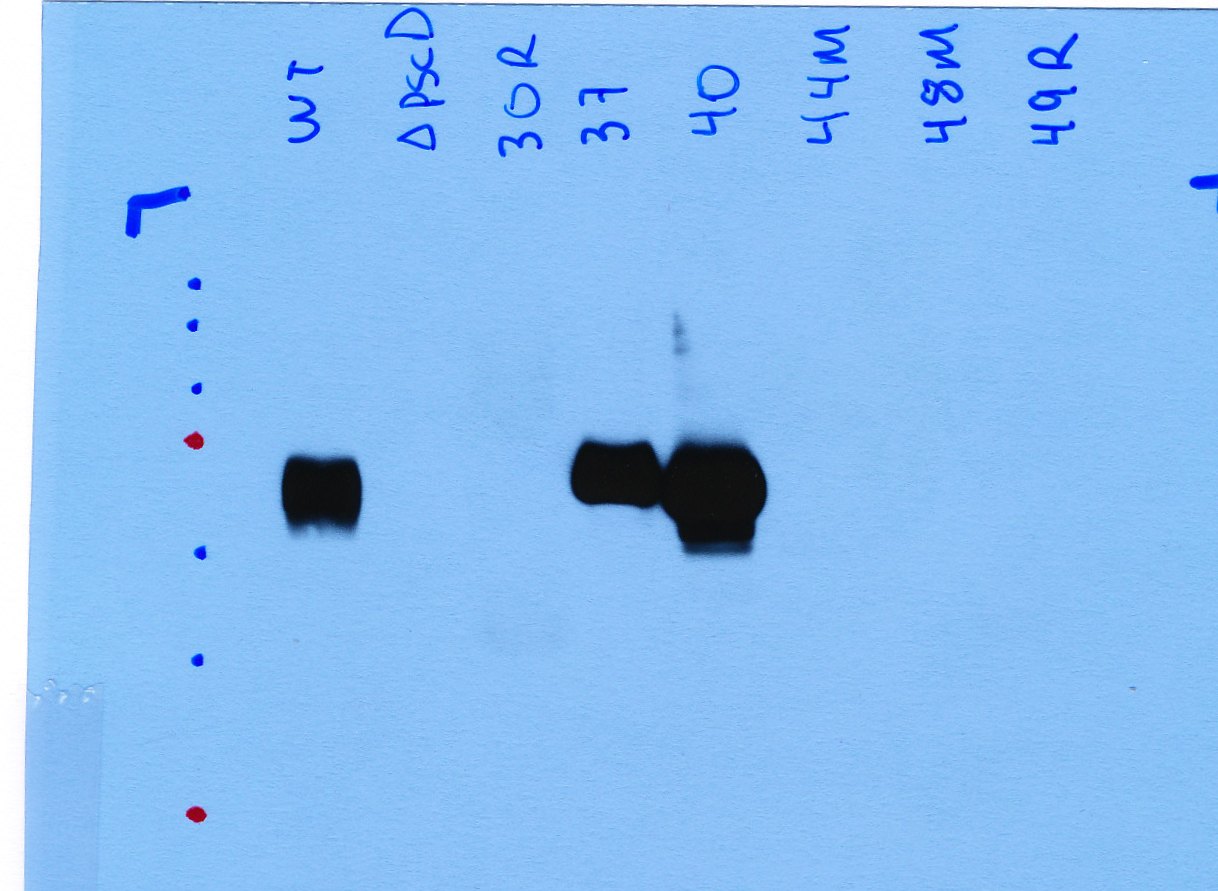

Supplement: Supplemental Information 4 [file peerj-13-19725-s004.zip › crude data and blots/Figure 1/Original blots/Duplicados/WB ExoU Fig 1.1.jpg]

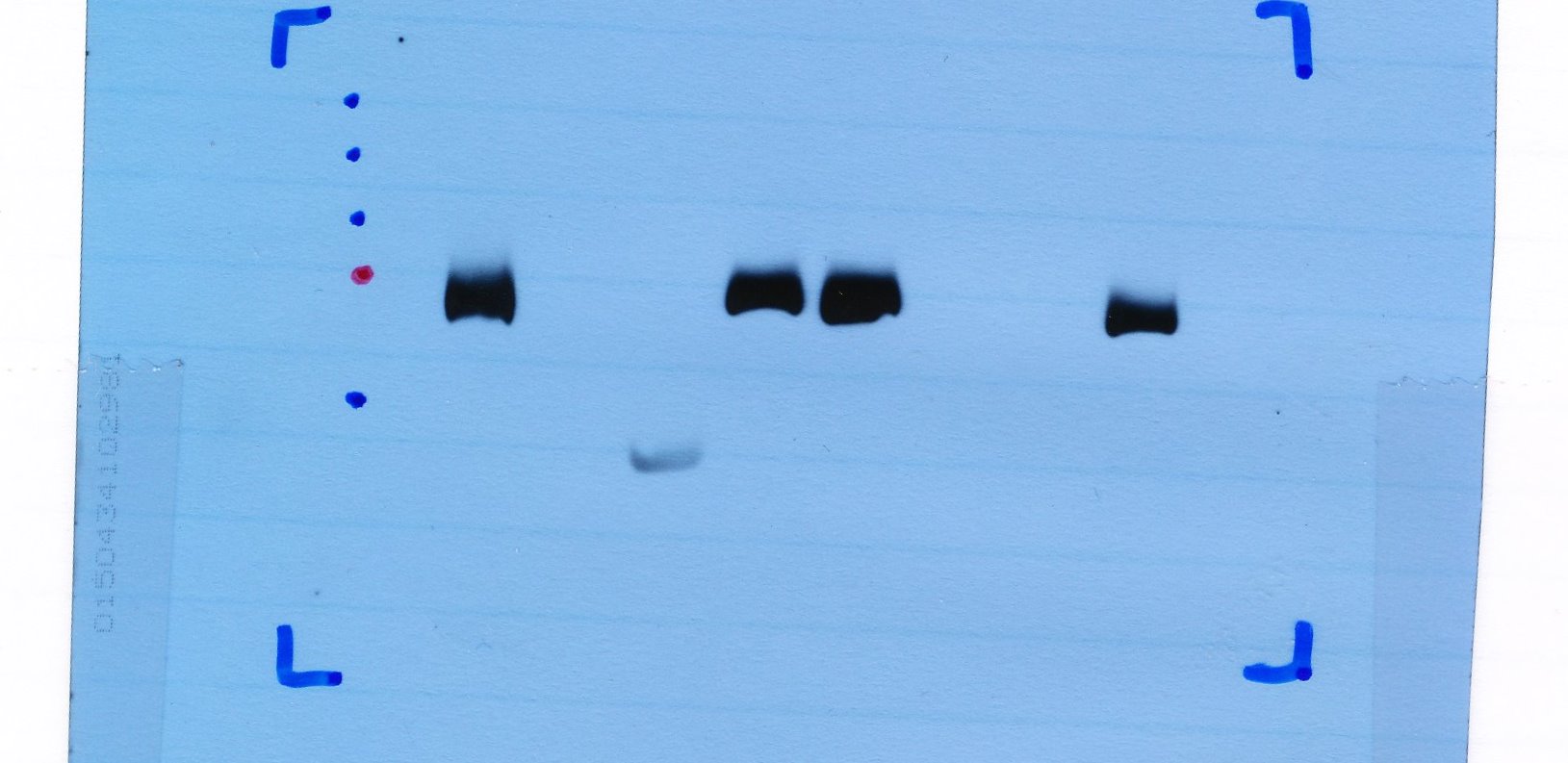

Supplement: Supplemental Information 4 [file peerj-13-19725-s004.zip › crude data and blots/Figure 1/Original blots/Duplicados/WB ExoU Fig 1.2.jpg]

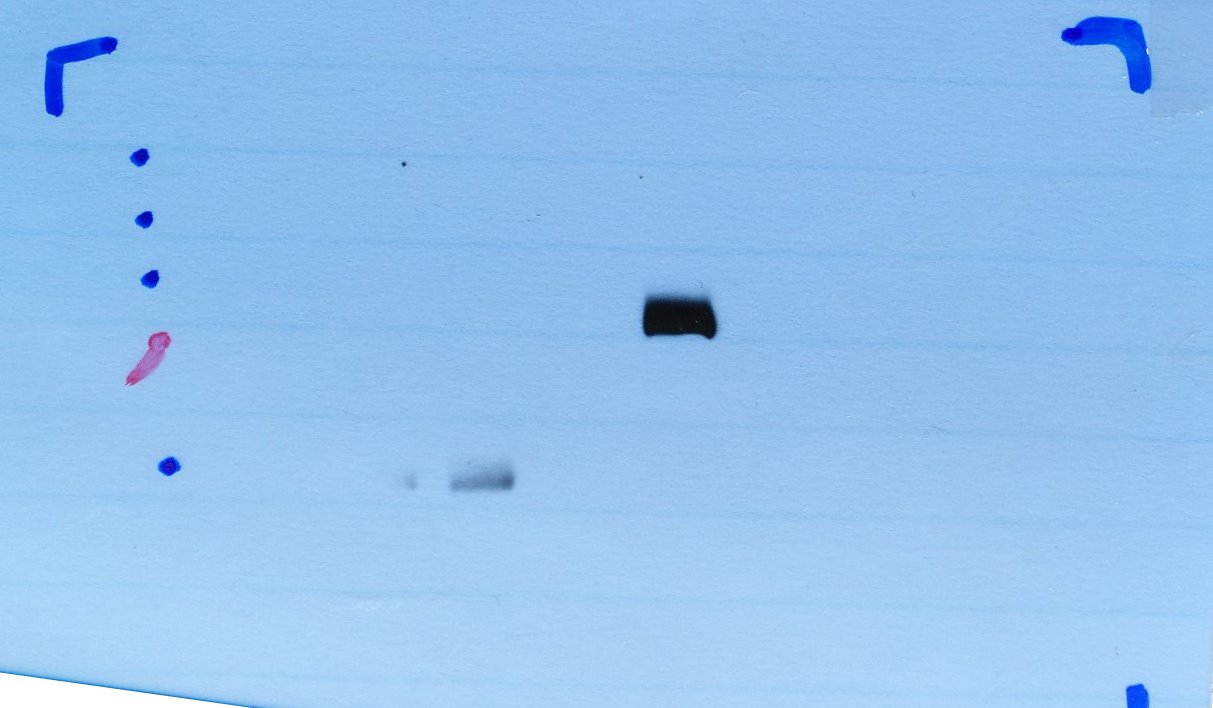

Supplement: Supplemental Information 4 [file peerj-13-19725-s004.zip › crude data and blots/Figure 1/Original blots/Duplicados/WB EXoU Fig 2.1.jpg]

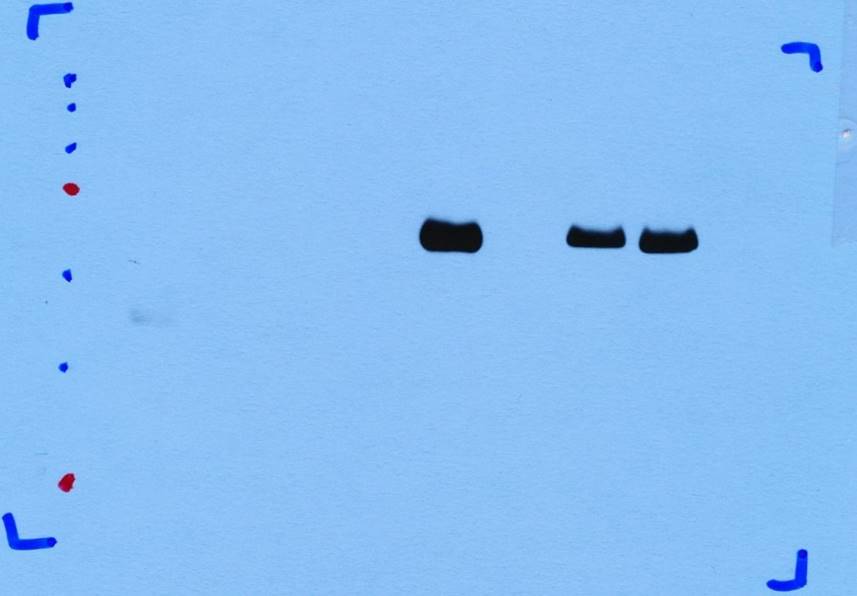

Supplement: Supplemental Information 4 [file peerj-13-19725-s004.zip › crude data and blots/Figure 1/Original blots/Duplicados/WB ExoU Fig 2.2.jpg]

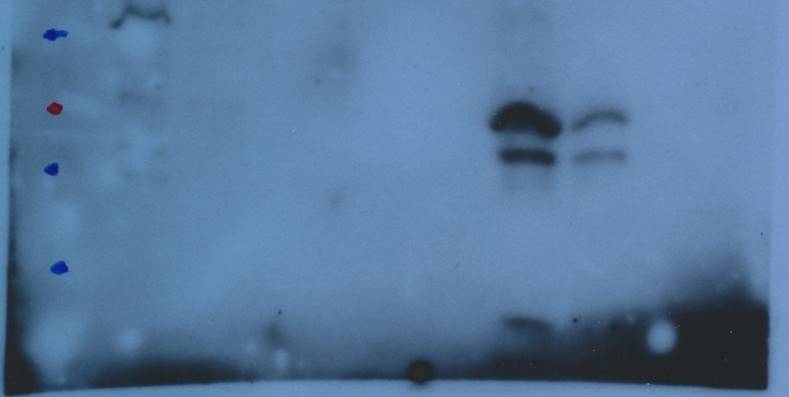

Supplement: Supplemental Information 4 [file peerj-13-19725-s004.zip › crude data and blots/Figure 1/Original blots/Duplicados/WB ExoU Fig 3.1.jpg]

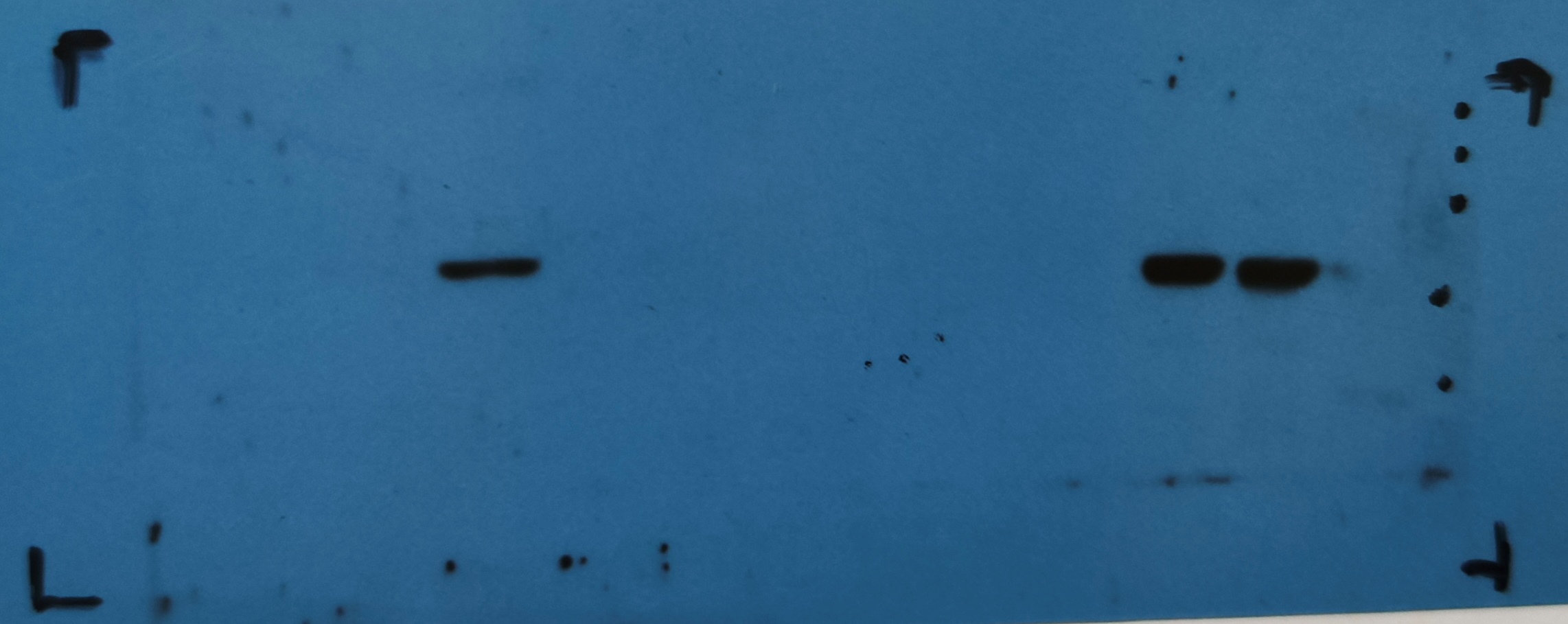

Supplement: Supplemental Information 4 [file peerj-13-19725-s004.zip › crude data and blots/Figure 1/Original blots/Duplicados/WB ExoU Fig 3.2.jpg]

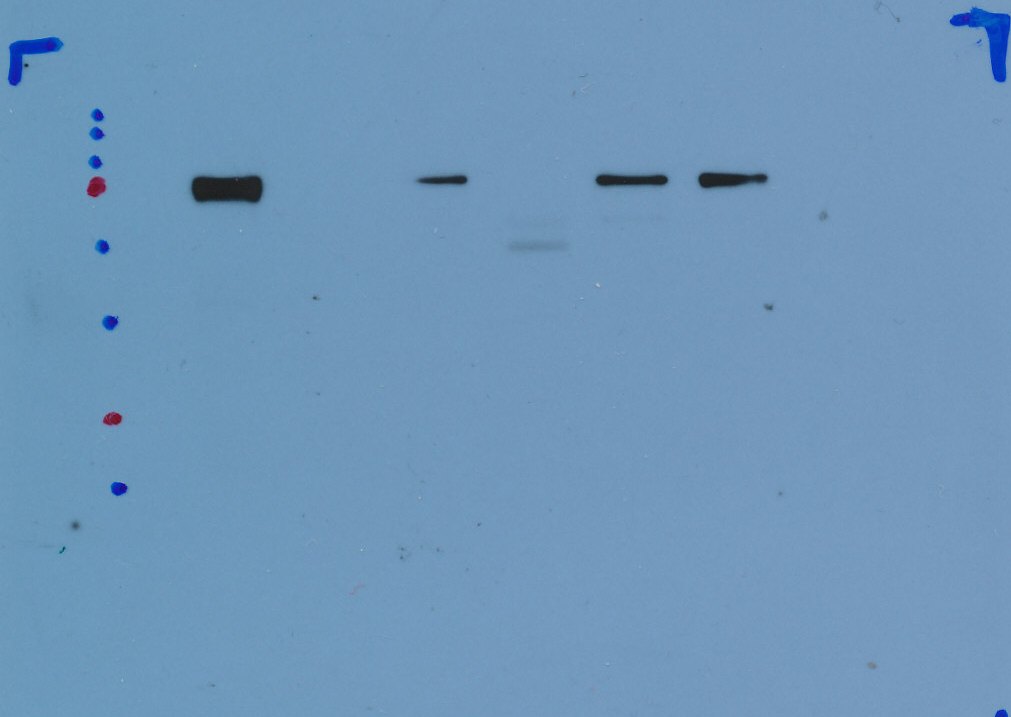

Supplement: Supplemental Information 4 [file peerj-13-19725-s004.zip › crude data and blots/Figure 1/Original blots/Duplicados/WB ExoU Fig 4.1.jpg]

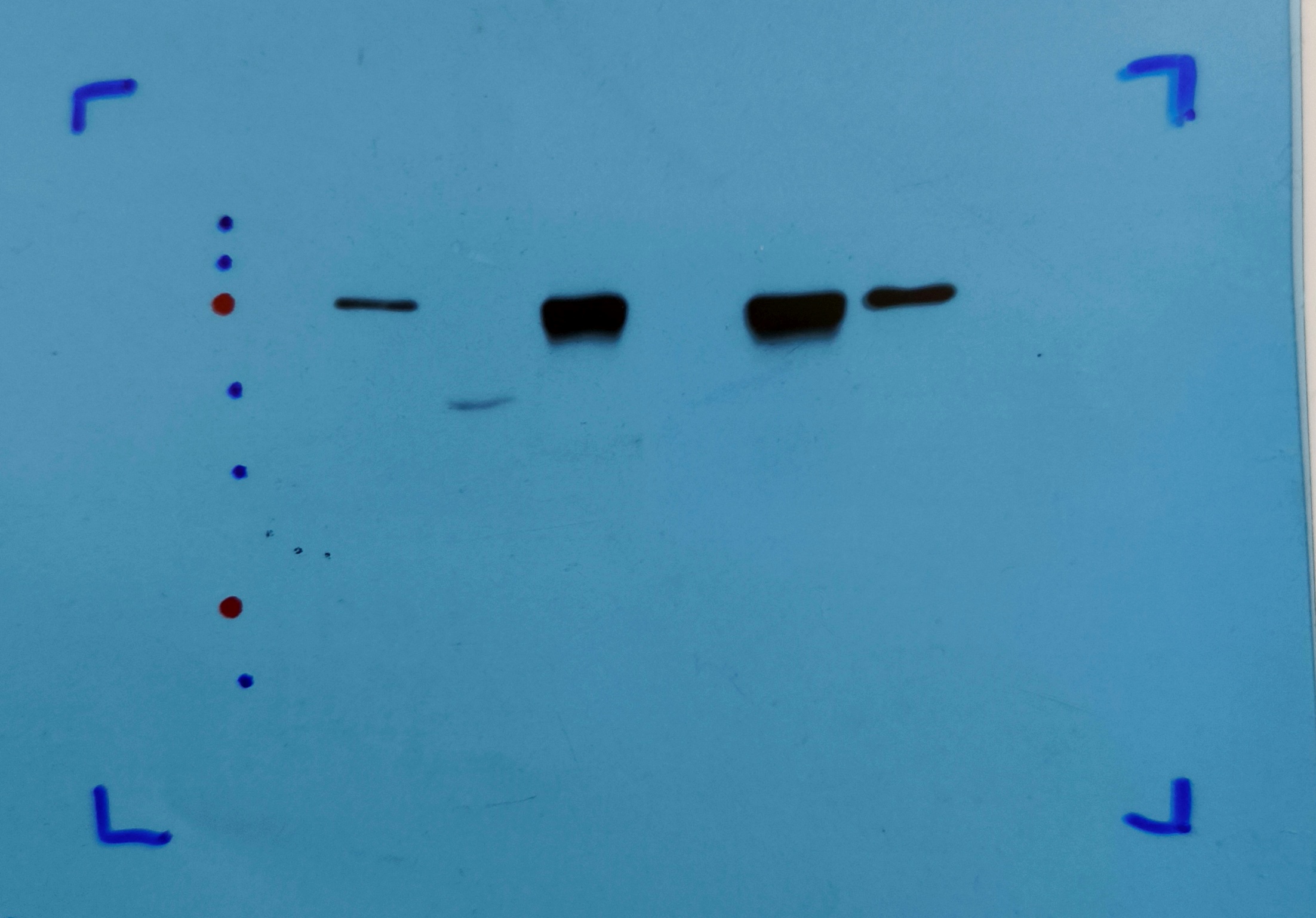

Supplement: Supplemental Information 4 [file peerj-13-19725-s004.zip › crude data and blots/Figure 1/Original blots/Duplicados/WB ExoU Fig4.2.jpg]

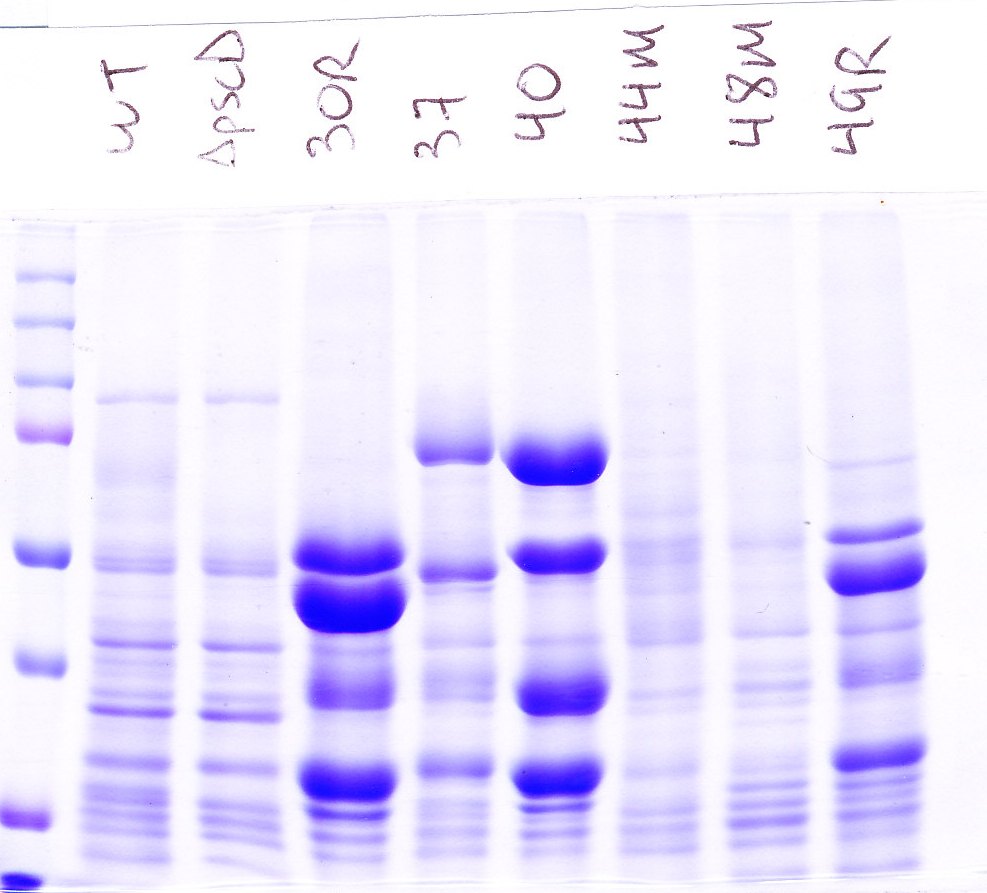

Supplement: Supplemental Information 4 [file peerj-13-19725-s004.zip › crude data and blots/Figure 1/Original blots/FIGURE 1/CBB FIG 1.jpg]

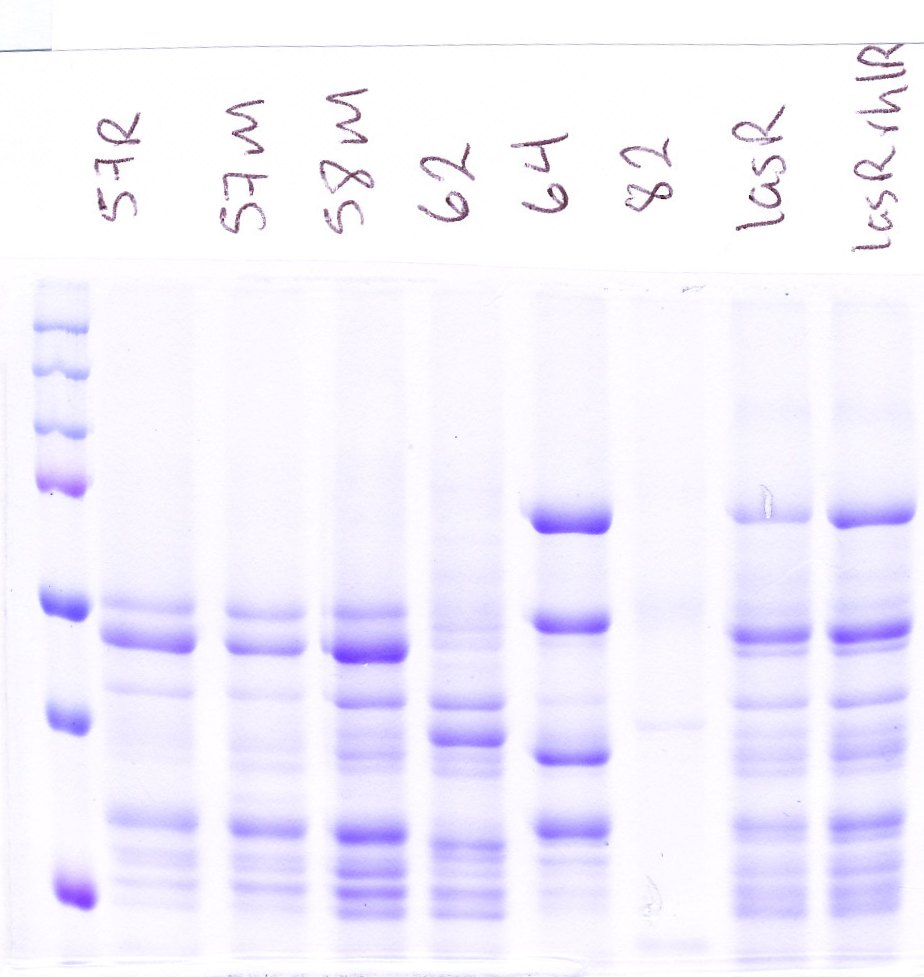

Supplement: Supplemental Information 4 [file peerj-13-19725-s004.zip › crude data and blots/Figure 1/Original blots/FIGURE 1/CBB FIG 2.jpg]

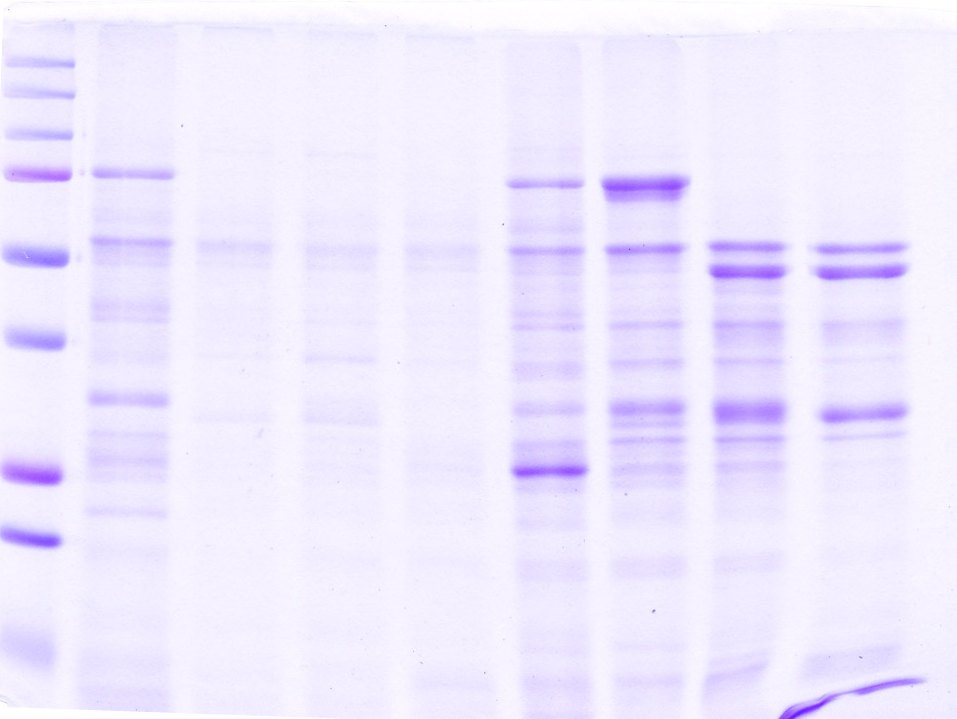

Supplement: Supplemental Information 4 [file peerj-13-19725-s004.zip › crude data and blots/Figure 1/Original blots/FIGURE 1/CBB FIG 3.jpg]

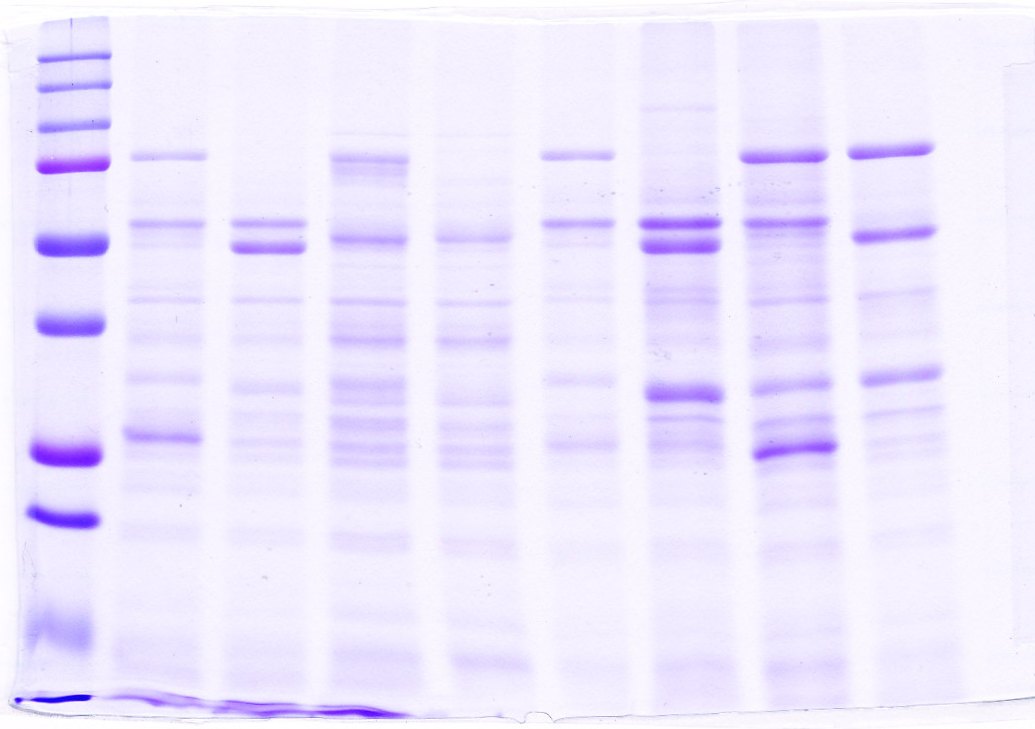

Supplement: Supplemental Information 4 [file peerj-13-19725-s004.zip › crude data and blots/Figure 1/Original blots/FIGURE 1/CBB FIG 4.jpg]

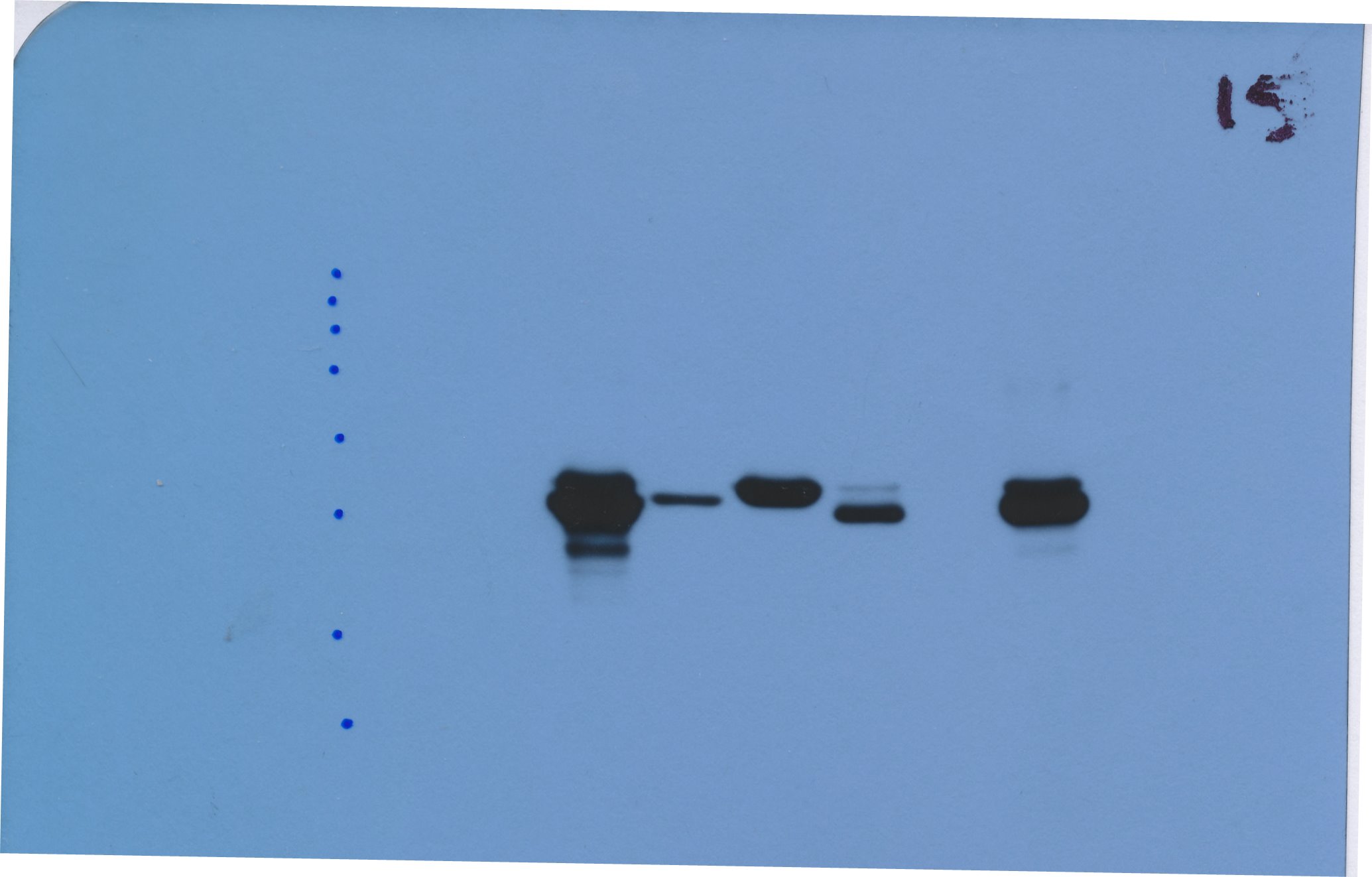

Supplement: Supplemental Information 4 [file peerj-13-19725-s004.zip › crude data and blots/Figure 1/Original blots/FIGURE 1/WB.ExoS 1.jpg]

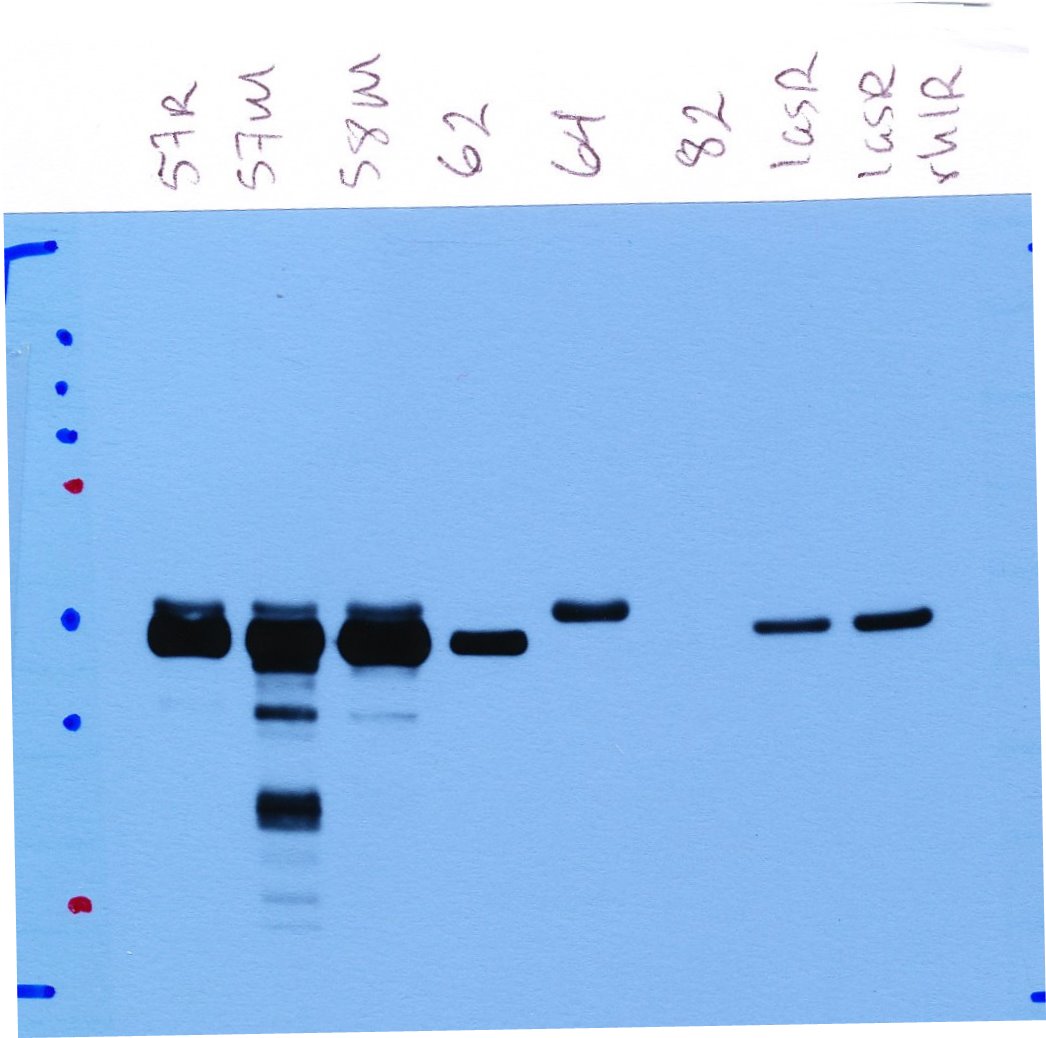

Supplement: Supplemental Information 4 [file peerj-13-19725-s004.zip › crude data and blots/Figure 1/Original blots/FIGURE 1/WB.ExoS 2.jpg]

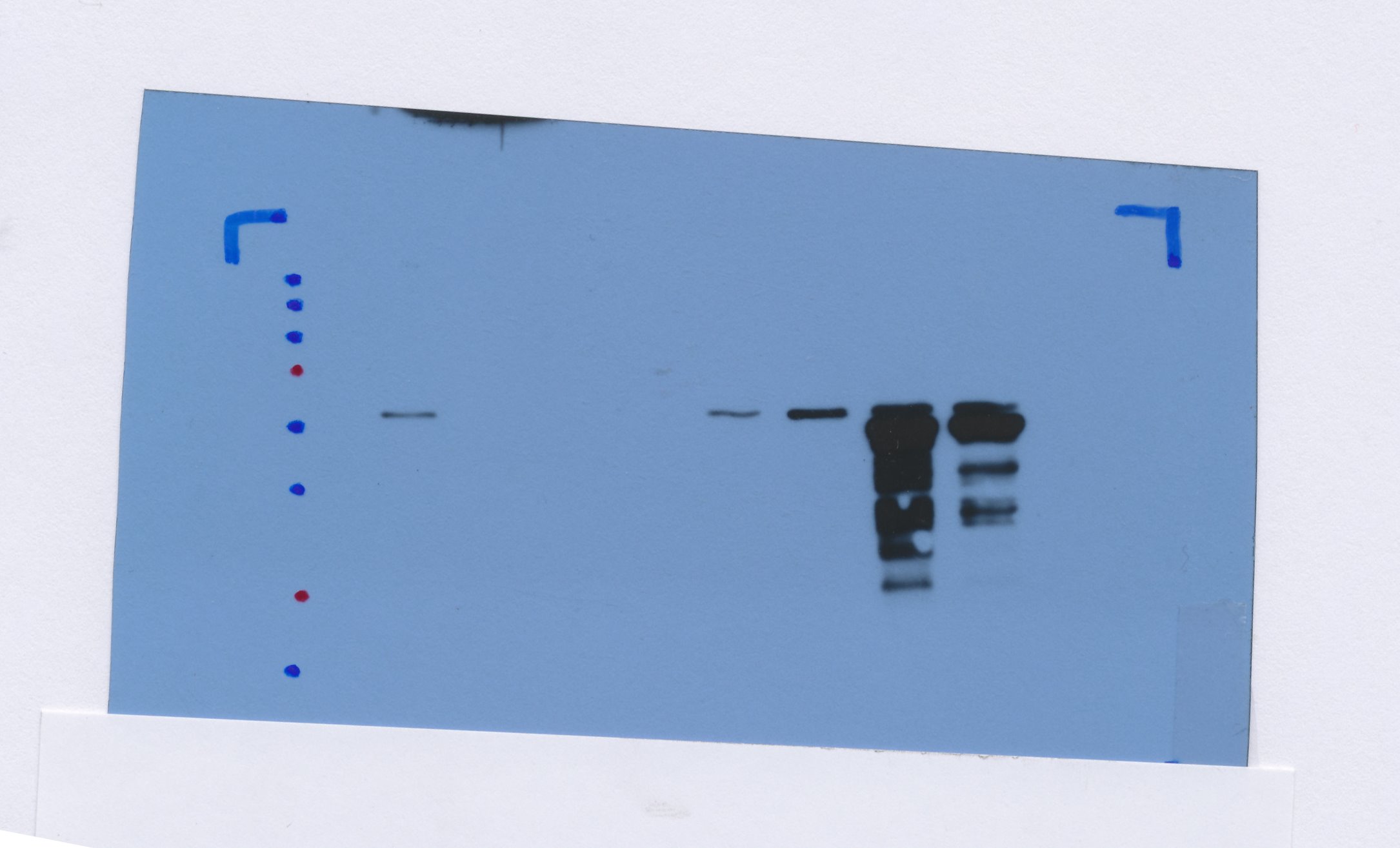

Supplement: Supplemental Information 4 [file peerj-13-19725-s004.zip › crude data and blots/Figure 1/Original blots/FIGURE 1/WB.ExoS 3.jpg]

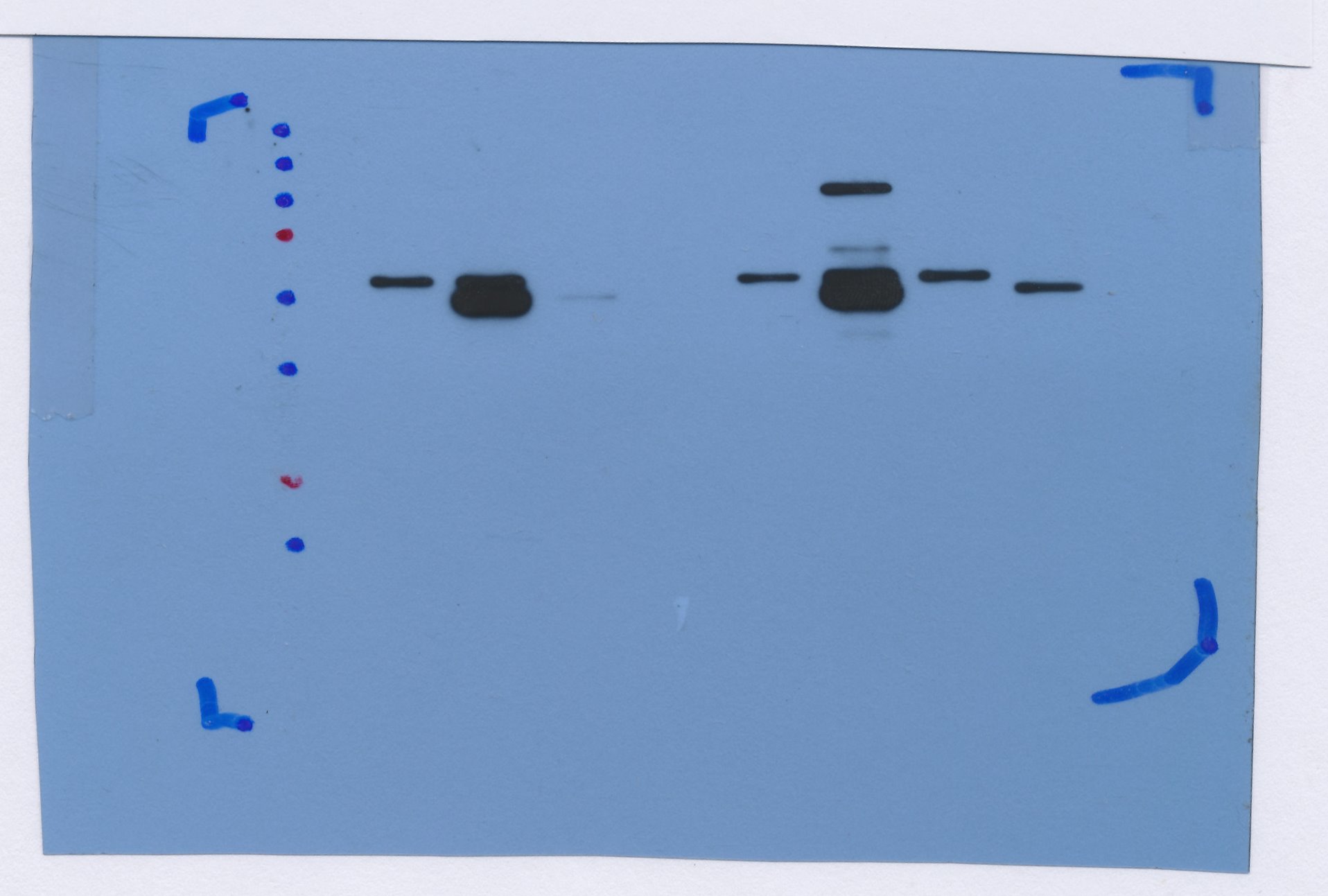

Supplement: Supplemental Information 4 [file peerj-13-19725-s004.zip › crude data and blots/Figure 1/Original blots/FIGURE 1/WB.ExoS 4.jpg]

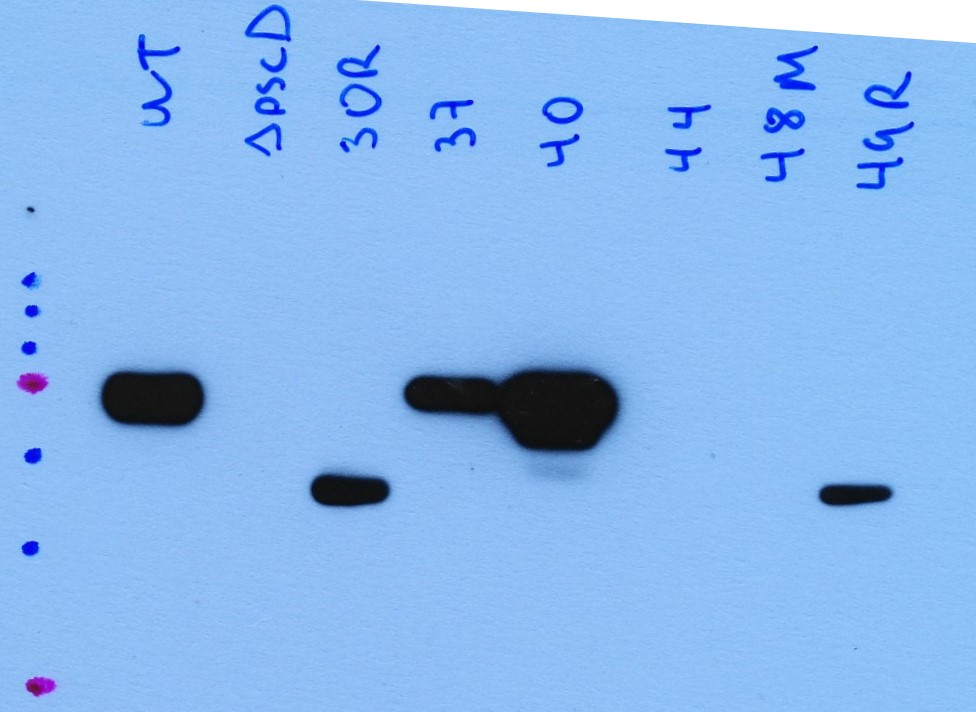

Supplement: Supplemental Information 4 [file peerj-13-19725-s004.zip › crude data and blots/Figure 1/Original blots/FIGURE 1/WB.ExoU 1.jpg]

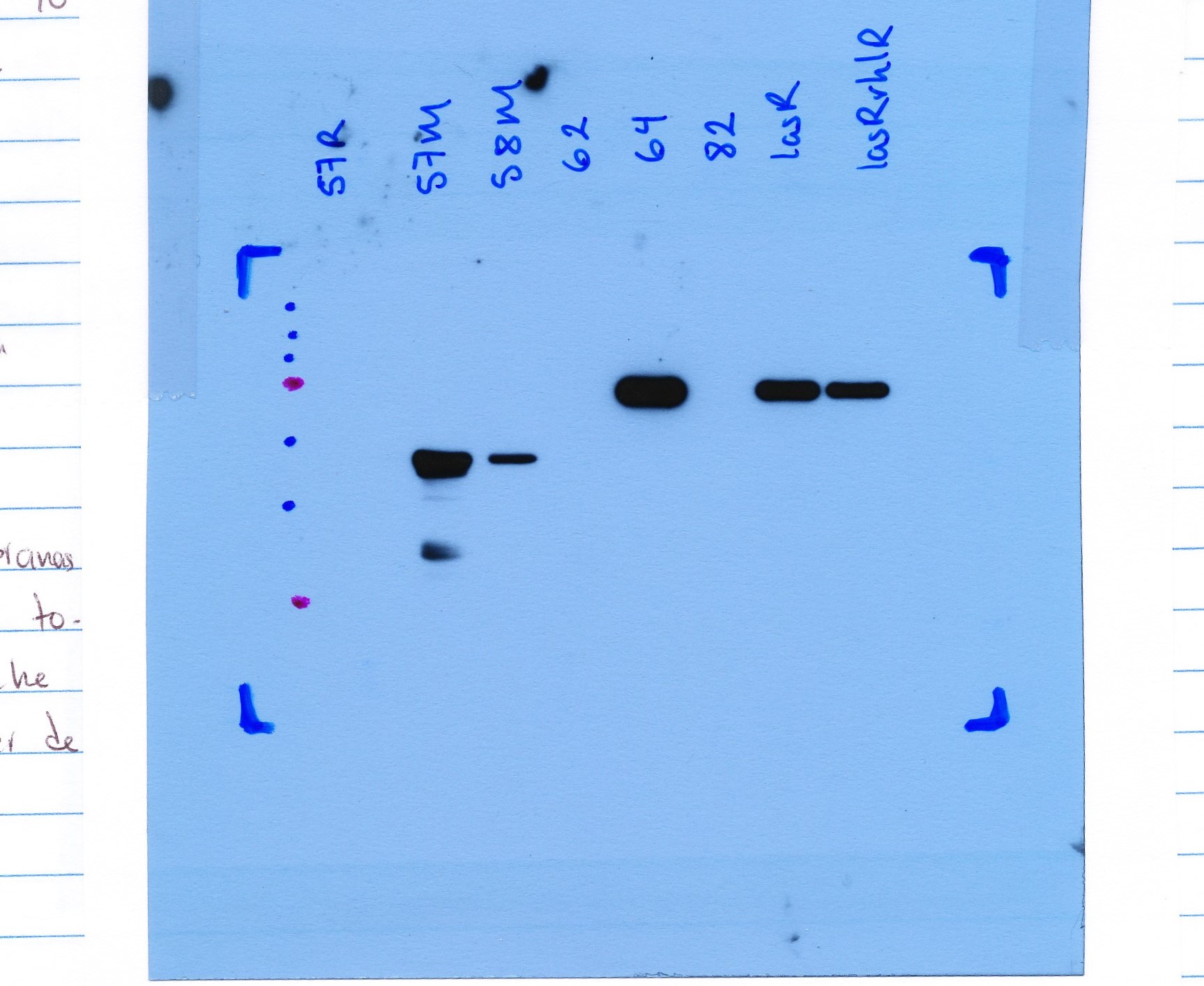

Supplement: Supplemental Information 4 [file peerj-13-19725-s004.zip › crude data and blots/Figure 1/Original blots/FIGURE 1/WB.ExoU 2.jpg]

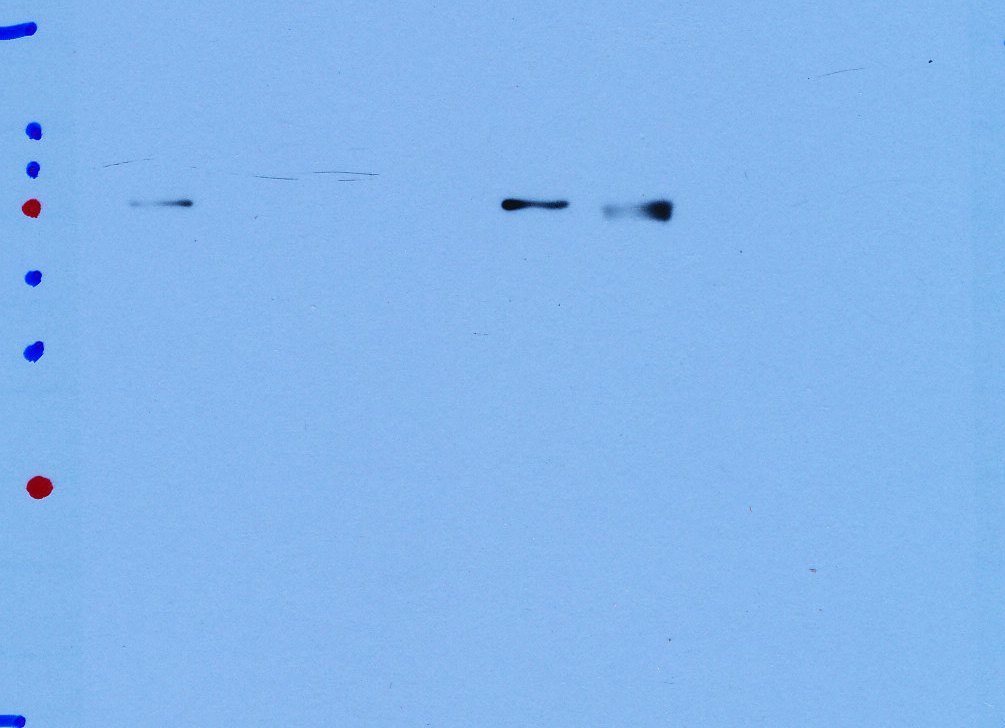

Supplement: Supplemental Information 4 [file peerj-13-19725-s004.zip › crude data and blots/Figure 1/Original blots/FIGURE 1/WB.ExoU 3.jpg]

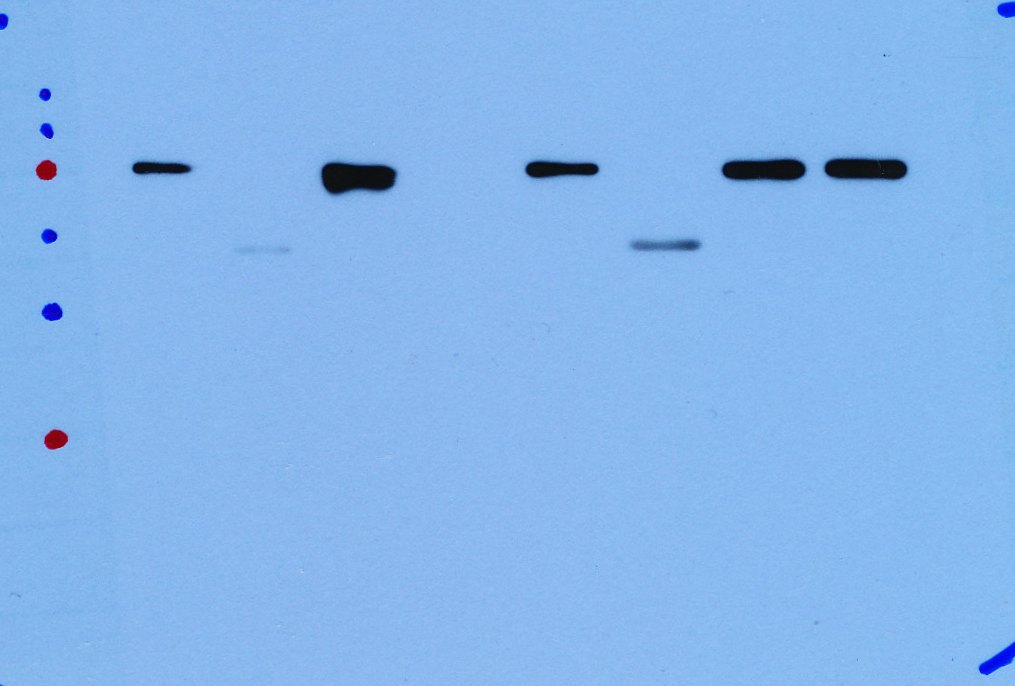

Supplement: Supplemental Information 4 [file peerj-13-19725-s004.zip › crude data and blots/Figure 1/Original blots/FIGURE 1/WB.ExoU 4.jpg]

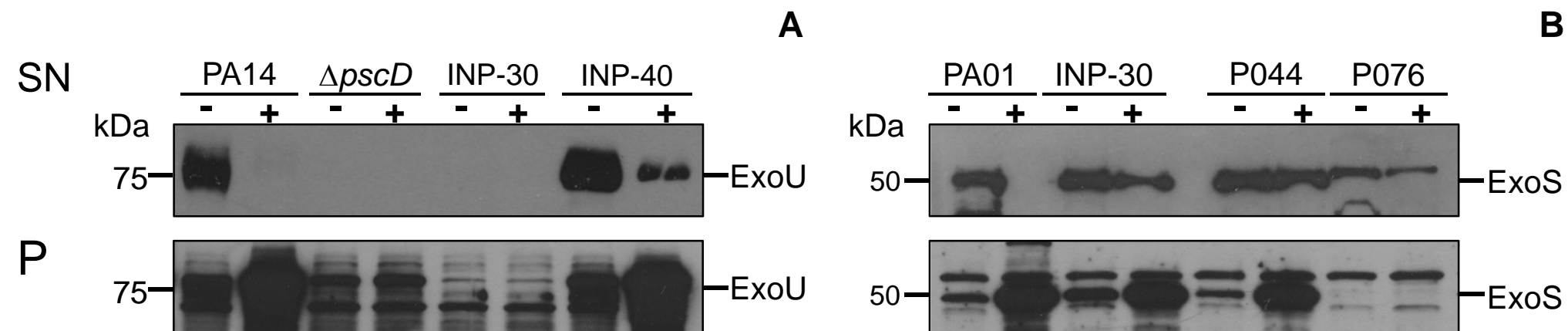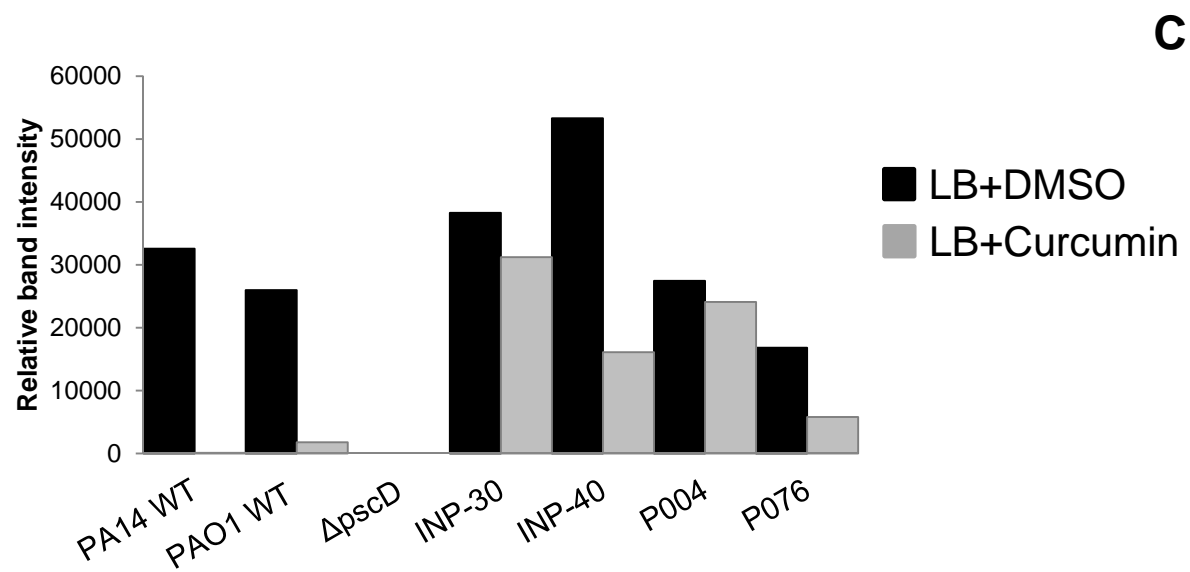

Supplement: Supplemental Information 4 [file peerj-13-19725-s004.zip › crude data and blots/Figure 2/Figure 2.pdf]

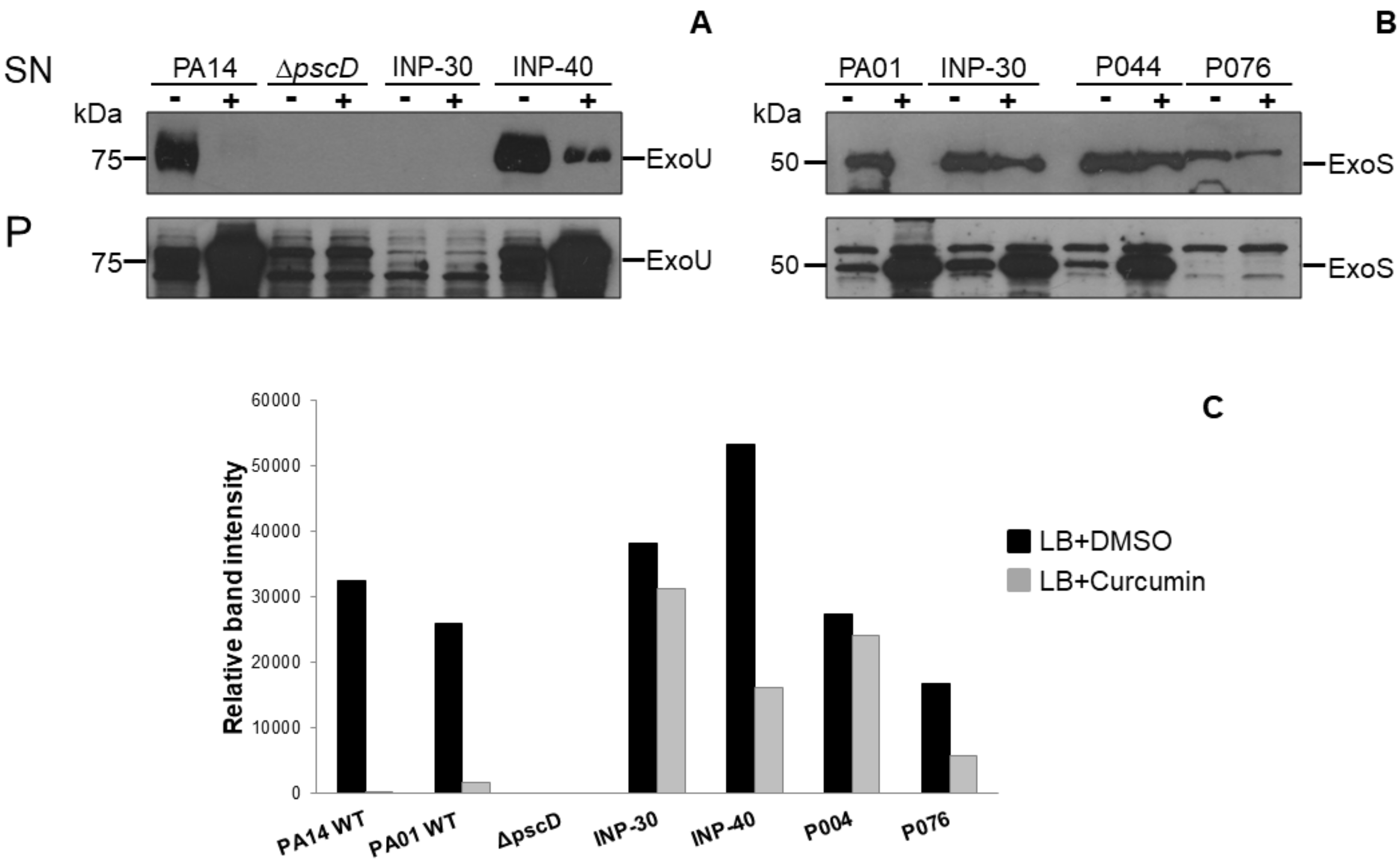

Supplement: Supplemental Information 4 [file peerj-13-19725-s004.zip › crude data and blots/Figure 2/Figure 2.tiff]

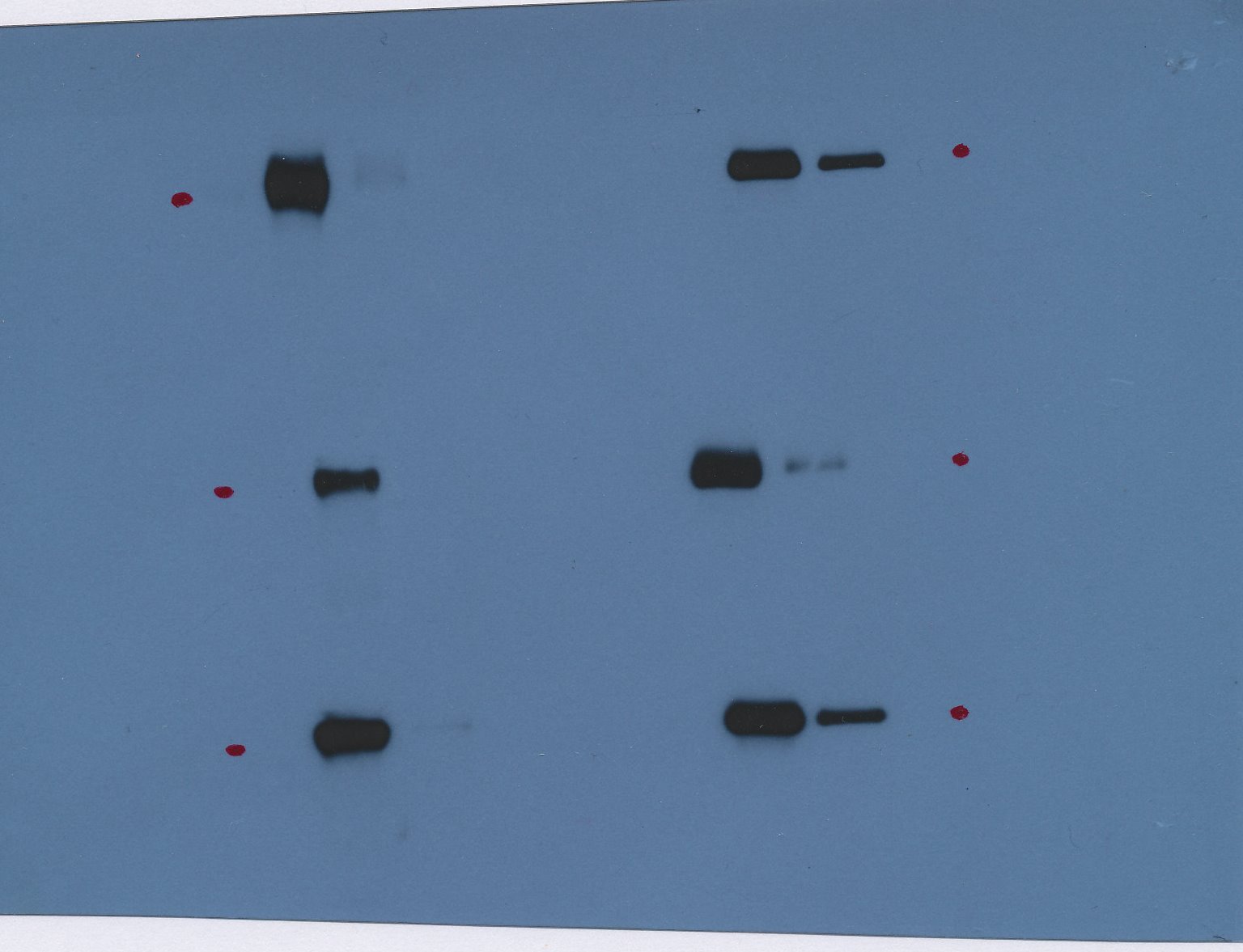

Supplement: Supplemental Information 4 [file peerj-13-19725-s004.zip › crude data and blots/Figure 2/Original blots/Duplicados/Fig 2 INP-40_ExoU.jpg]

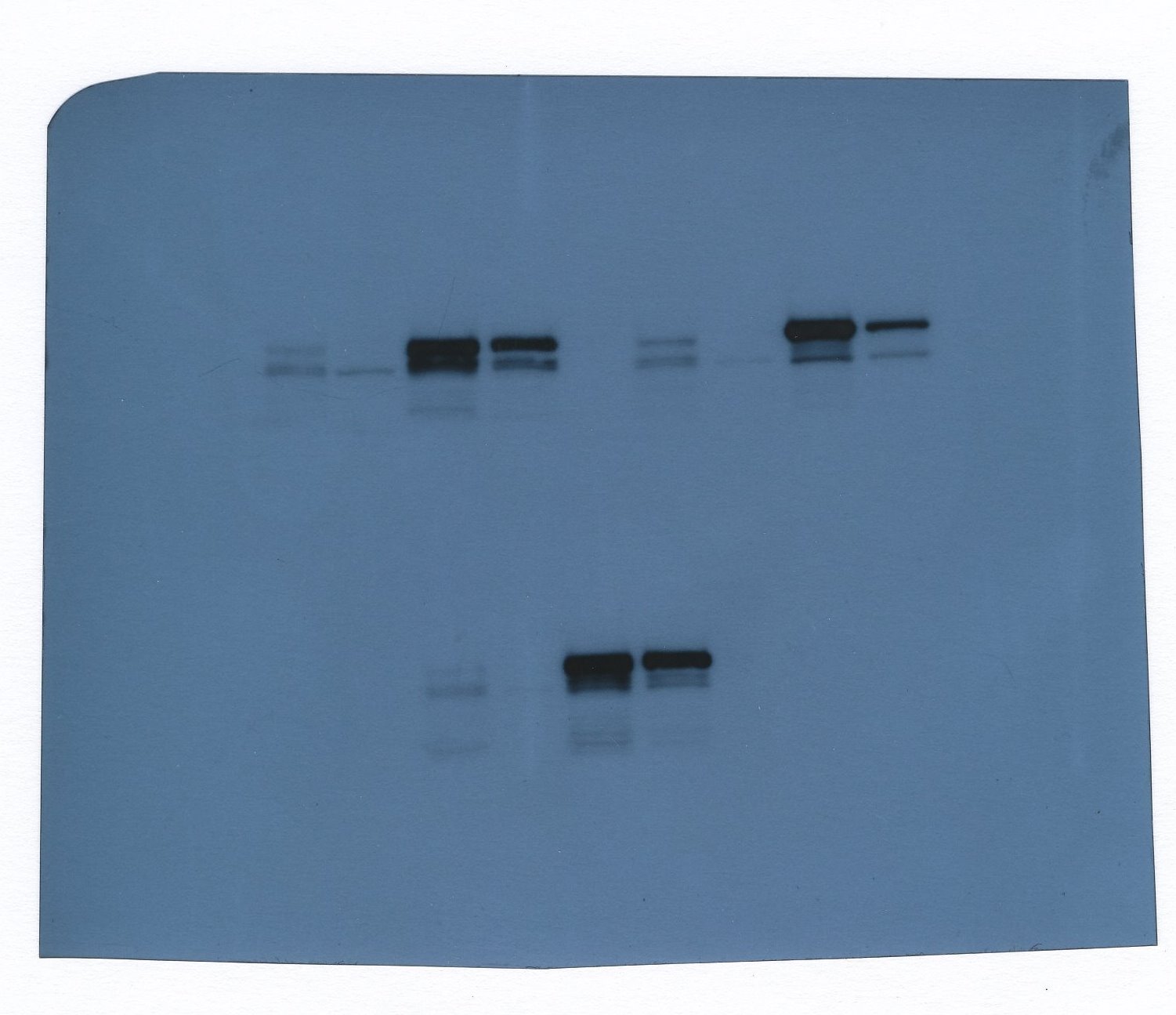

Supplement: Supplemental Information 4 [file peerj-13-19725-s004.zip › crude data and blots/Figure 2/Original blots/Duplicados/Fig 2 P193 SN_ExoU.jpg]

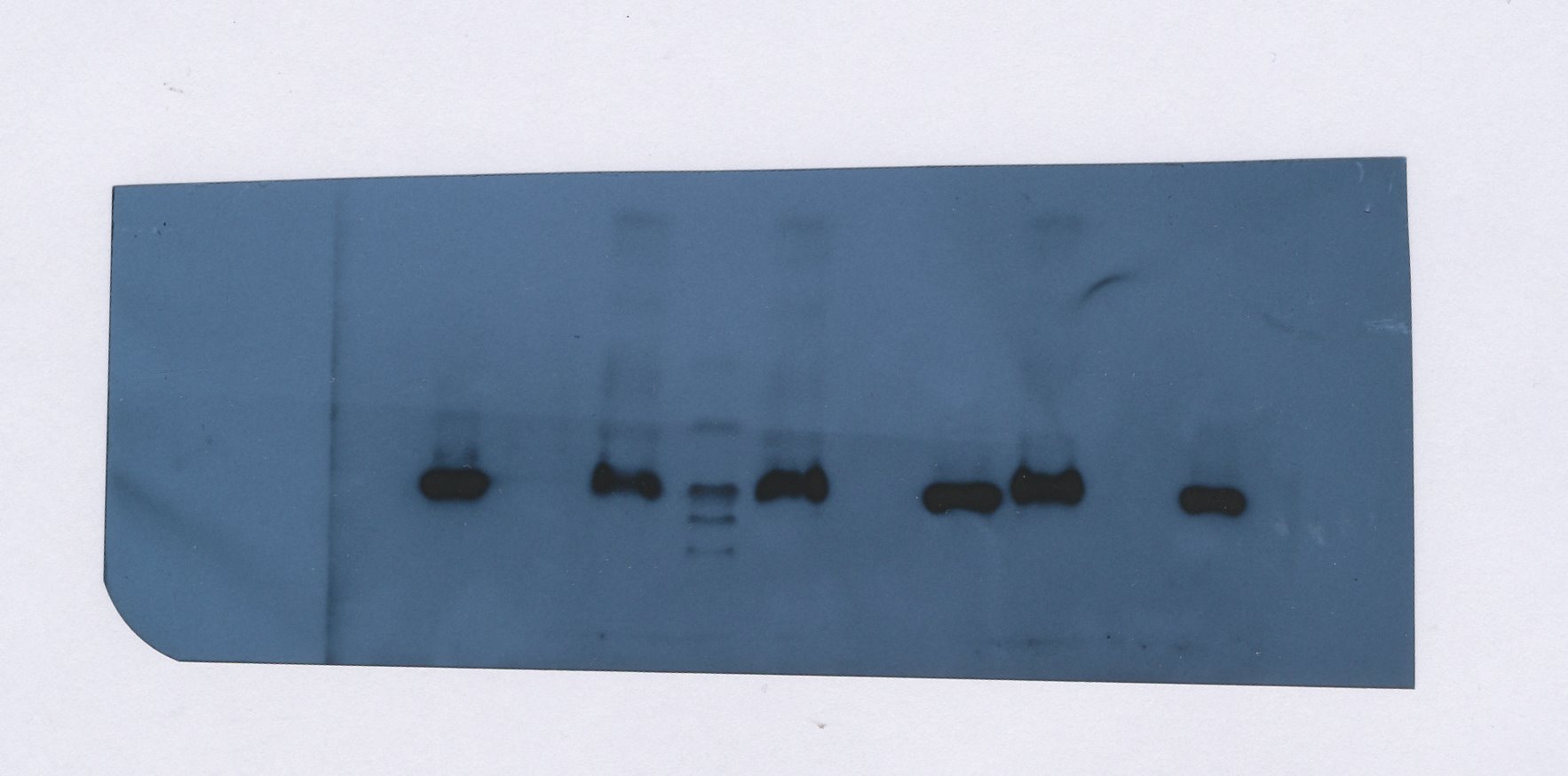

Supplement: Supplemental Information 4 [file peerj-13-19725-s004.zip › crude data and blots/Figure 2/Original blots/Duplicados/Fig 2. SN_P044_ExoS.jpg]

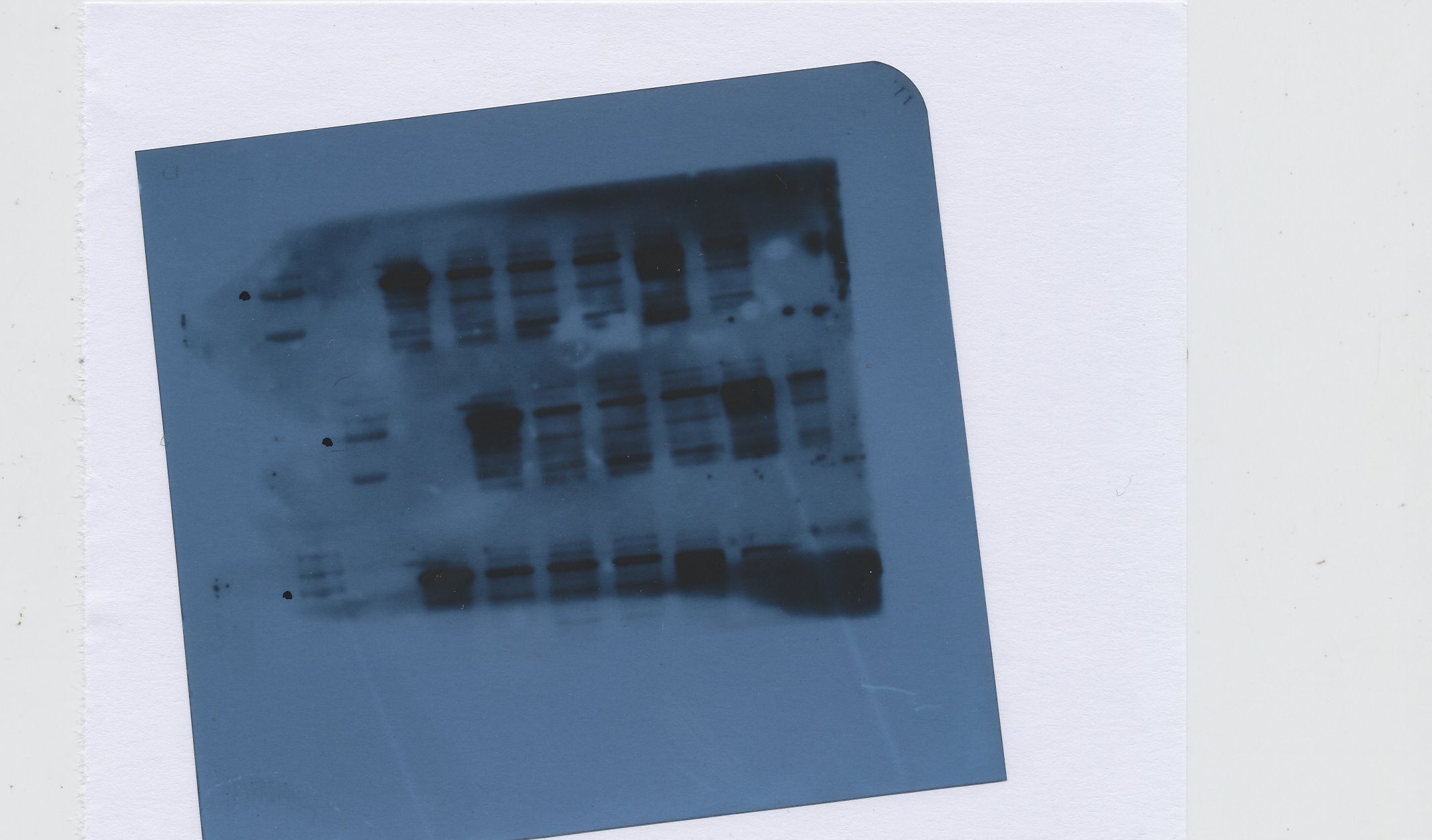

Supplement: Supplemental Information 4 [file peerj-13-19725-s004.zip › crude data and blots/Figure 2/Original blots/Duplicados/Fig 2_P_P076_ExoU.jpg]

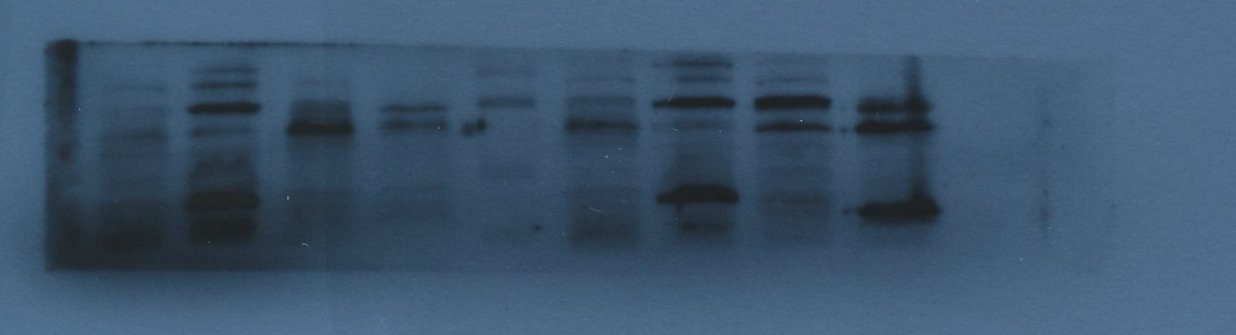

Supplement: Supplemental Information 4 [file peerj-13-19725-s004.zip › crude data and blots/Figure 2/Original blots/Duplicados/Fig 2_P_P193_ExoU.jpg]

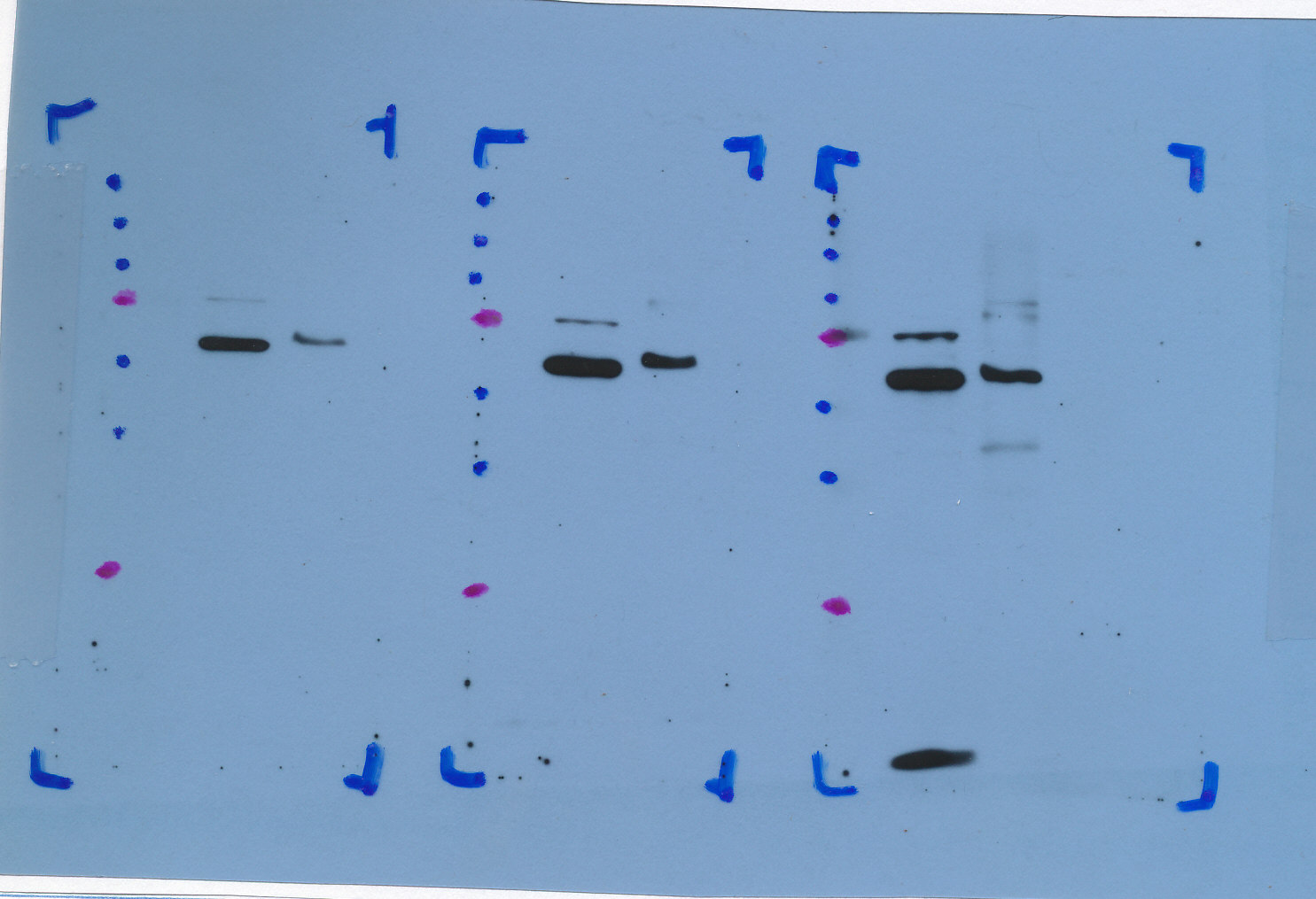

Supplement: Supplemental Information 4 [file peerj-13-19725-s004.zip › crude data and blots/Figure 2/Original blots/Duplicados/Fig 2_SN_P076_ExoS.jpg]

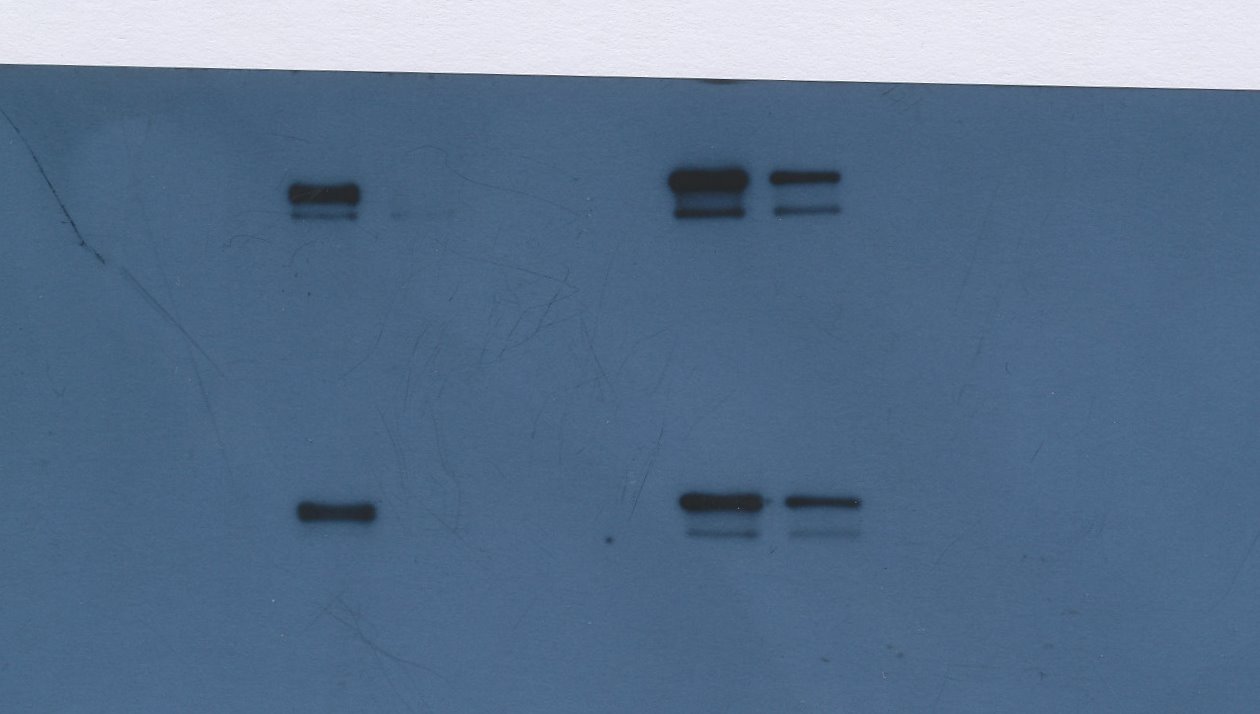

Supplement: Supplemental Information 4 [file peerj-13-19725-s004.zip › crude data and blots/Figure 2/Original blots/Duplicados/Fig 2_SN_P076_ExoU.jpg]

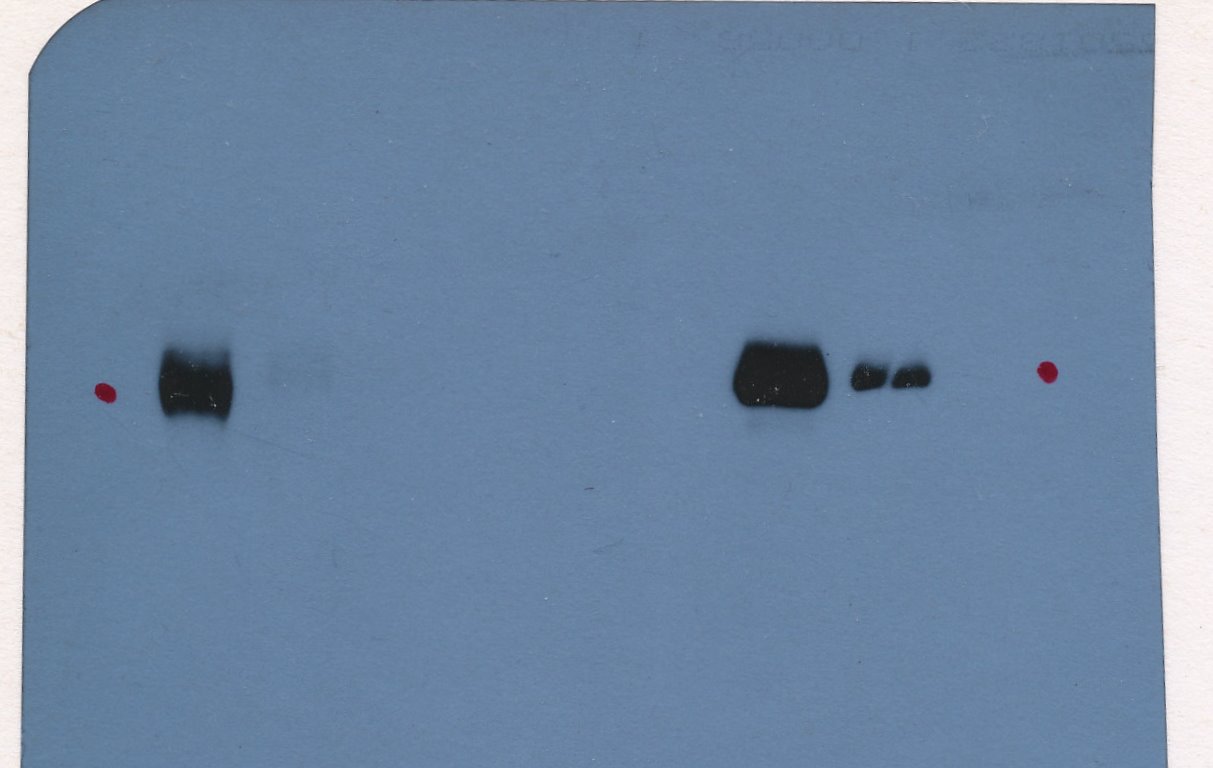

Supplement: Supplemental Information 4 [file peerj-13-19725-s004.zip › crude data and blots/Figure 2/Original blots/Fig 2_1.jpg]

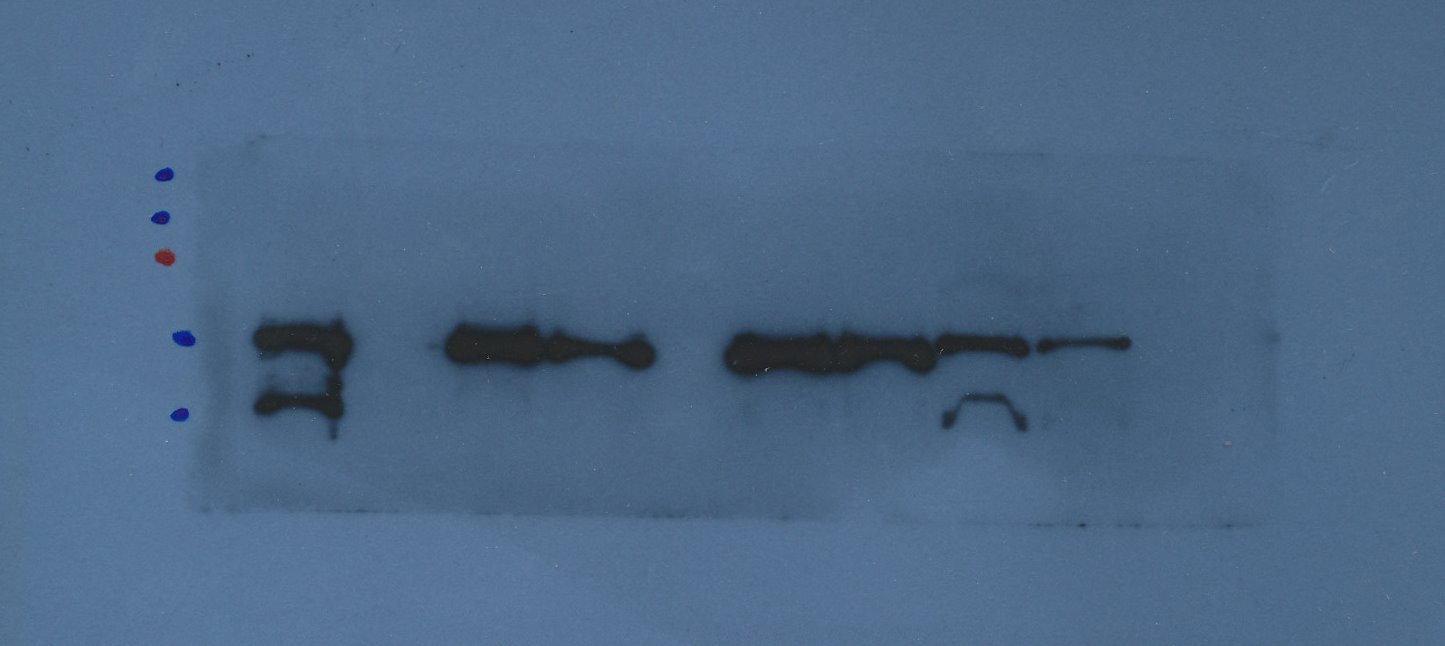

Supplement: Supplemental Information 4 [file peerj-13-19725-s004.zip › crude data and blots/Figure 2/Original blots/Fig 2_2.jpg]

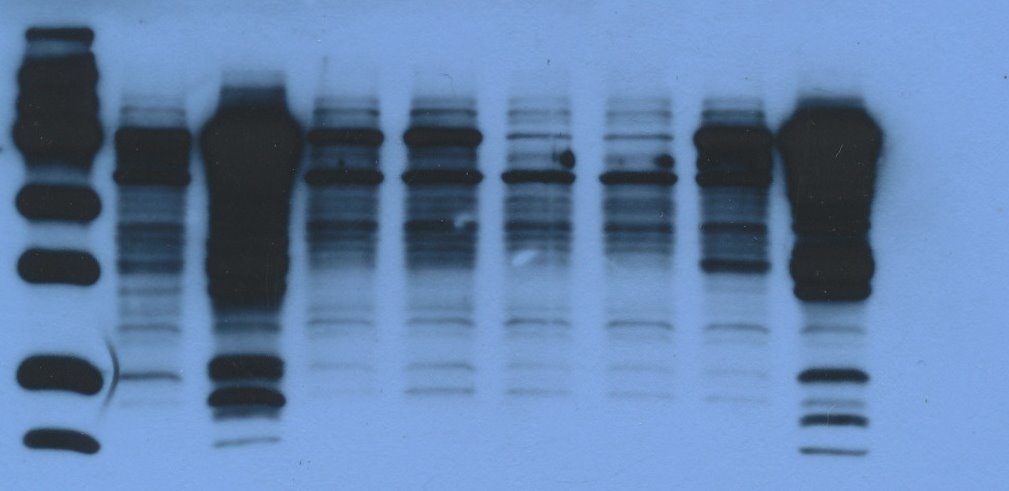

Supplement: Supplemental Information 4 [file peerj-13-19725-s004.zip › crude data and blots/Figure 2/Original blots/Fig 2_3.jpg]

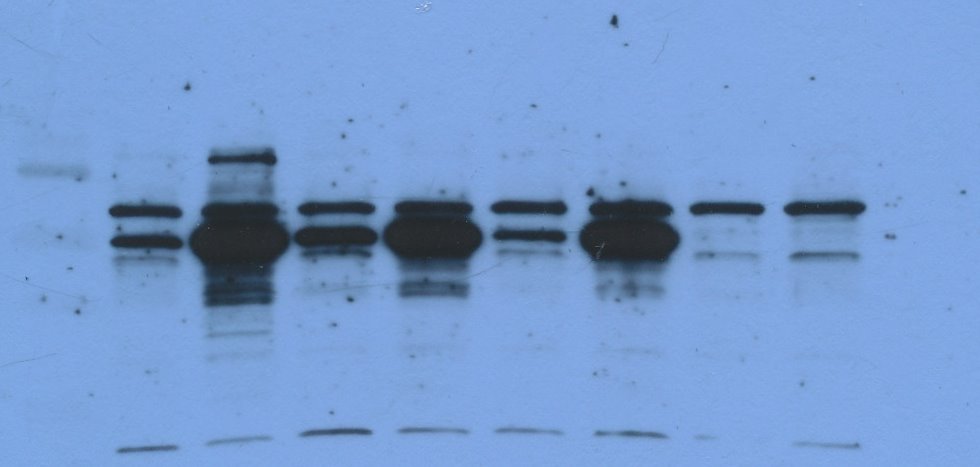

Supplement: Supplemental Information 4 [file peerj-13-19725-s004.zip › crude data and blots/Figure 2/Original blots/Fig 2_4.jpg]

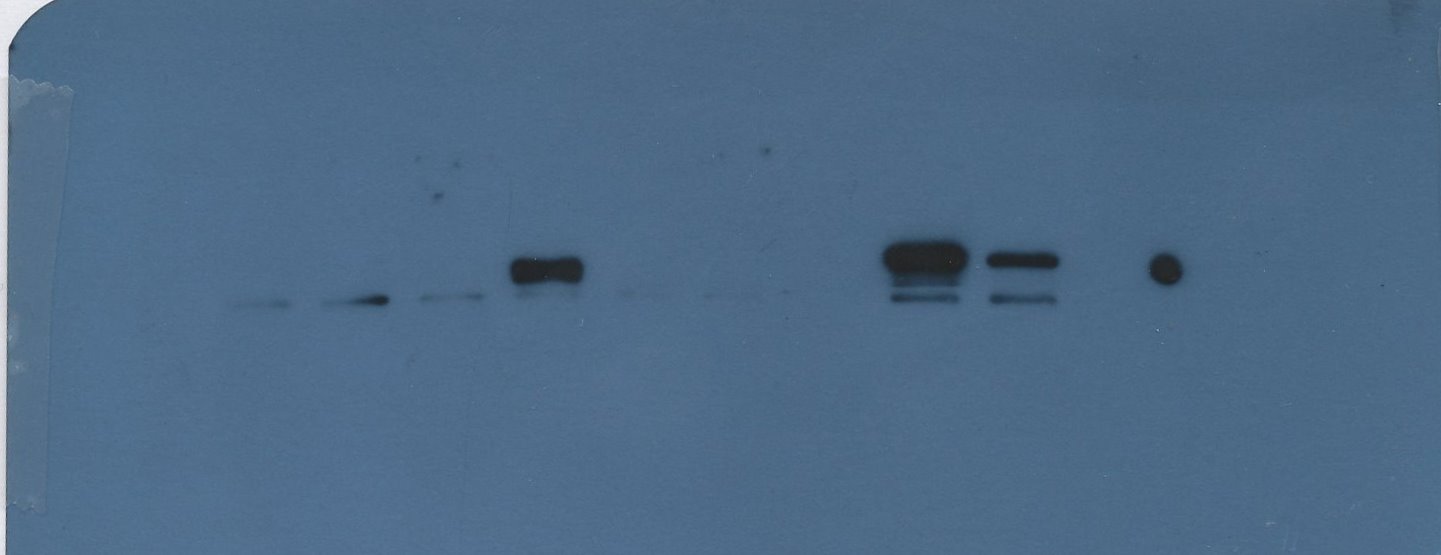

Supplement: Supplemental Information 4 [file peerj-13-19725-s004.zip › crude data and blots/Figure 2/Original blots/Fig 2_5.jpg]

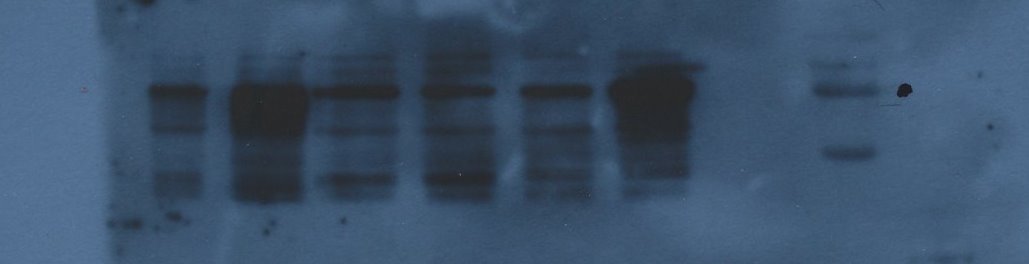

Supplement: Supplemental Information 4 [file peerj-13-19725-s004.zip › crude data and blots/Figure 2/Original blots/Fig 2_6 .jpg]

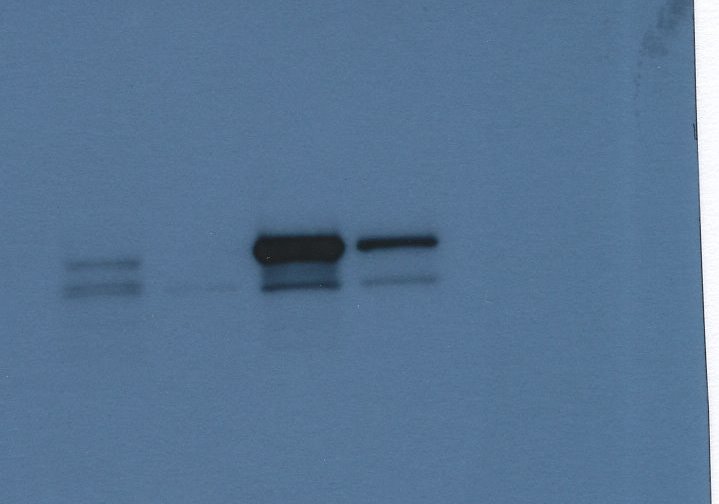

Supplement: Supplemental Information 4 [file peerj-13-19725-s004.zip › crude data and blots/Figure 2/Original blots/Fig 2_7.jpg]

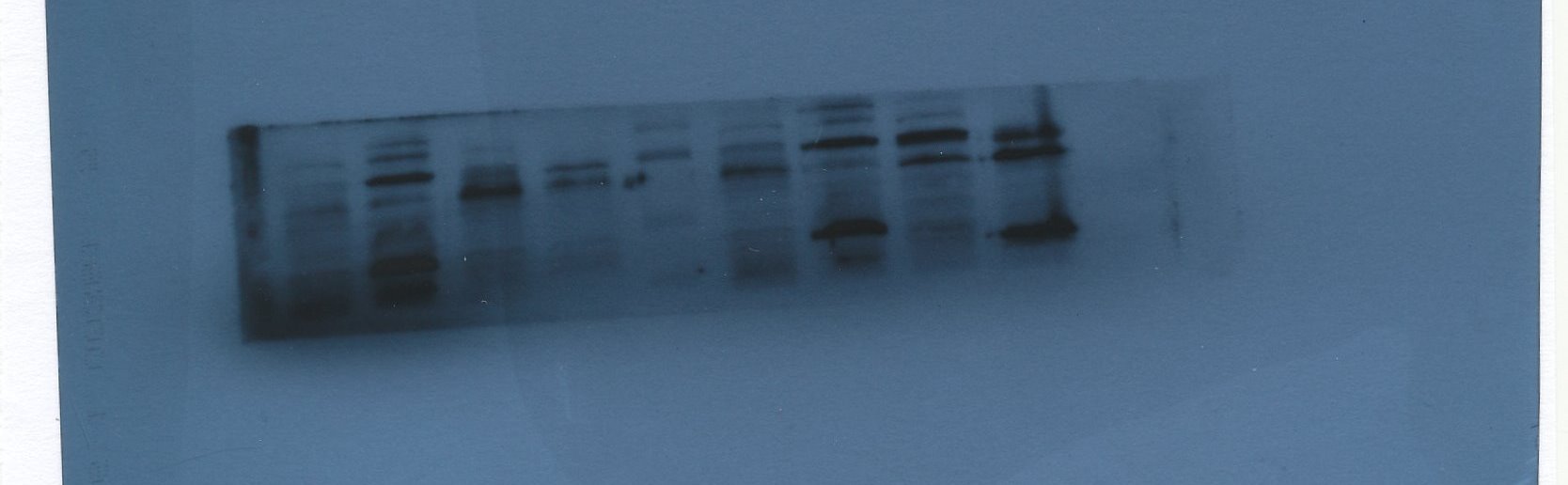

Supplement: Supplemental Information 4 [file peerj-13-19725-s004.zip › crude data and blots/Figure 2/Original blots/Fig 2_8.jpg]

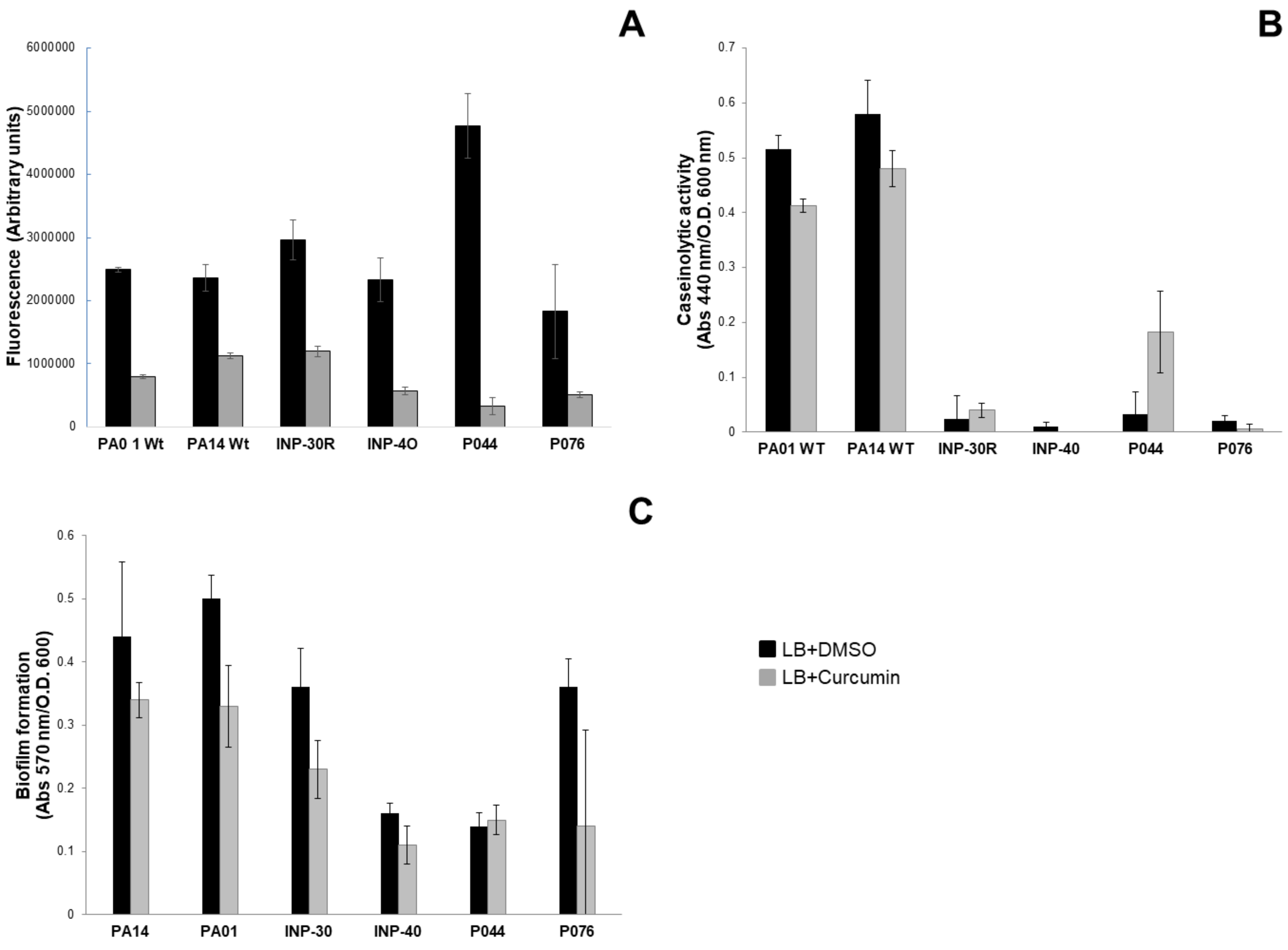

Supplement: Supplemental Information 4 [file peerj-13-19725-s004.zip › crude data and blots/Figure S2/Figure 3.Final.tiff]

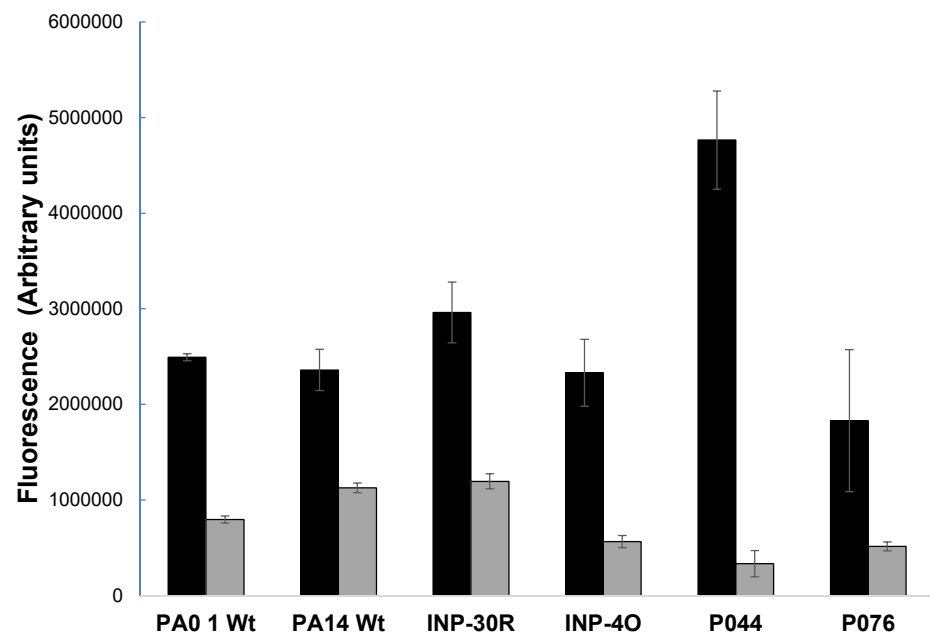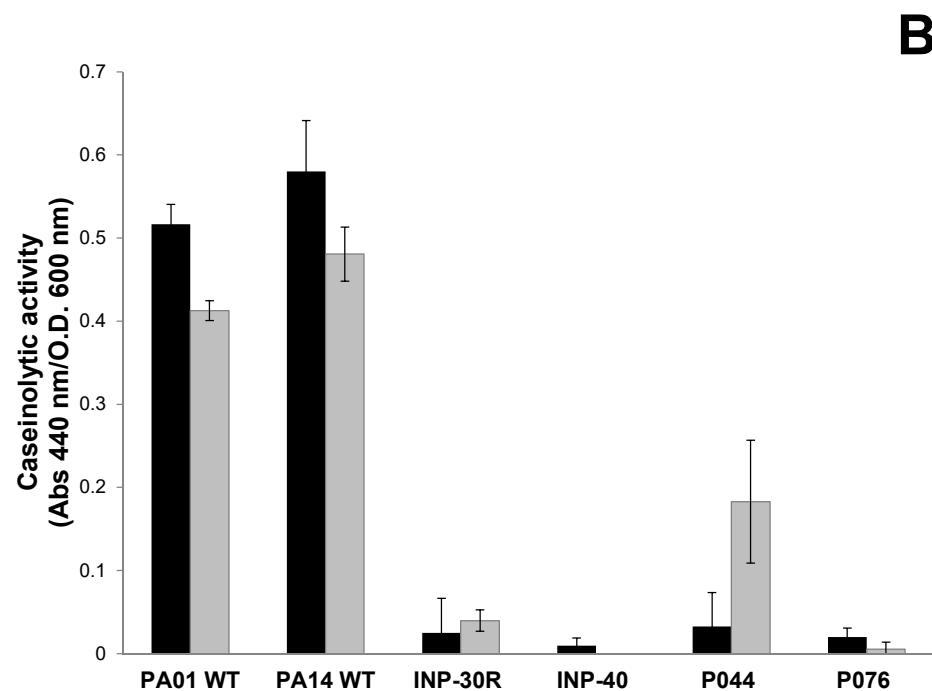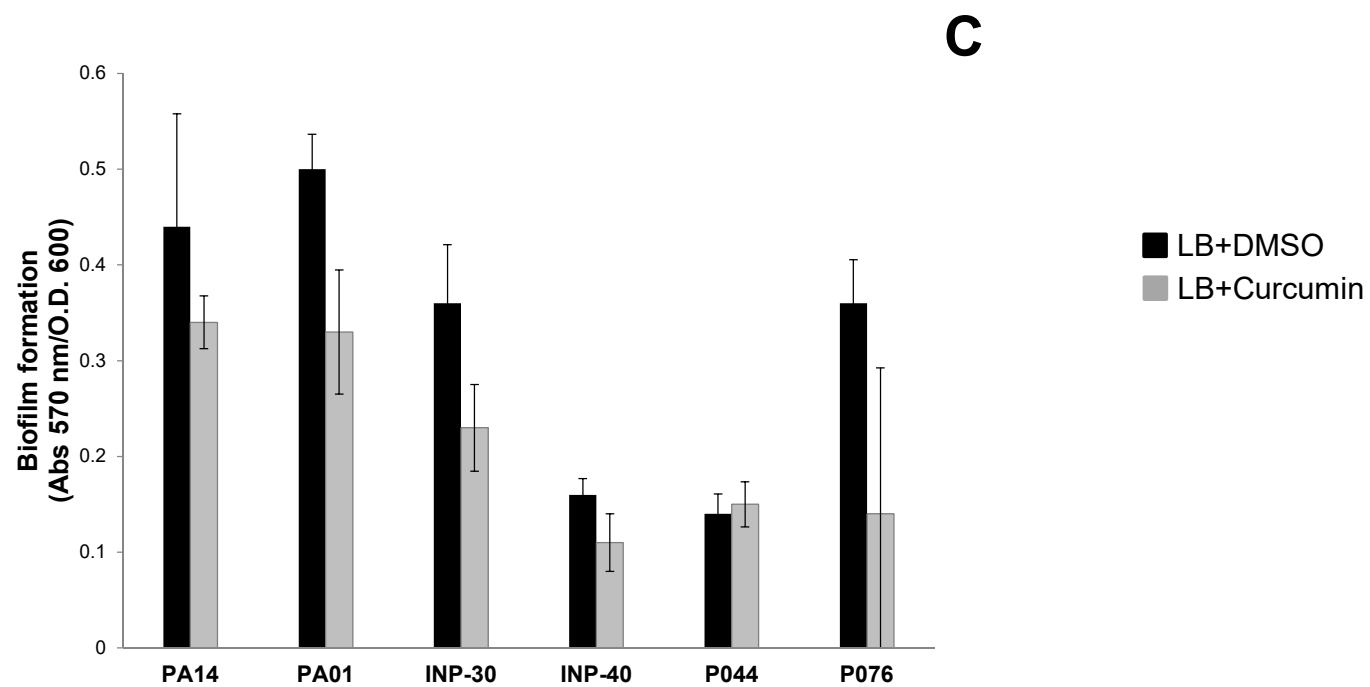

Supplement: Supplemental Information 4 [file peerj-13-19725-s004.zip › crude data and blots/Figure S2/Figure 3.pdf]
